# Sample multiplexing-based targeted pathway proteomics with real-time analytics reveals the impact of genetic variation on protein expression

**DOI:** 10.1038/s41467-023-36269-7

**Published:** 2023-02-02

**Authors:** Qing Yu, Xinyue Liu, Mark P. Keller, Jose Navarrete-Perea, Tian Zhang, Sipei Fu, Laura P. Vaites, Steven R. Shuken, Ernst Schmid, Gregory R. Keele, Jiaming Li, Edward L. Huttlin, Edrees H. Rashan, Judith Simcox, Gary A. Churchill, Devin K. Schweppe, Alan D. Attie, Joao A. Paulo, Steven P. Gygi

**Affiliations:** 1 Department of Cell Biology, Harvard Medical School, Boston, MA 02115 USA; 2 Department of Biochemistry, University of Wisconsin-Madison, Madison, WI 53706 USA; 3 Department of Biological Chemistry and Molecular Pharmacology, Harvard Medical School, Boston, MA 02115 USA; 4 The Jackson Laboratory, Bar Harbor, Maine 04609 USA; 5 Department of Genome Sciences, University of Washington, Seattle, WA 98105 USA

**Keywords:** Proteomics, Mass spectrometry, Proteome informatics, Quantitative trait

## Abstract

Targeted proteomics enables hypothesis-driven research by measuring the cellular expression of protein cohorts related by function, disease, or class after perturbation. Here, we present a pathway-centric approach and an assay builder resource for targeting entire pathways of up to 200 proteins selected from >10,000 expressed proteins to directly measure their abundances, exploiting sample multiplexing to increase throughput by 16-fold. The strategy, termed GoDig, requires only a single-shot LC-MS analysis, ~1 µg combined peptide material, a list of up to 200 proteins, and real-time analytics to trigger simultaneous quantification of up to 16 samples for hundreds of analytes. We apply GoDig to quantify the impact of genetic variation on protein expression in mice fed a high-fat diet. We create several GoDig assays to quantify the expression of multiple protein families (kinases, lipid metabolism- and lipid droplet-associated proteins) across 480 fully-genotyped Diversity Outbred mice, revealing protein quantitative trait loci and establishing potential linkages between specific proteins and lipid homeostasis.

## Abstract (web summary)

Targeted proteomics enables robust hypothesis-driven research. Here, Yu *et al*. present a multiplexed approach for targeted pathway proteomics and apply it to quantify protein families across 480 fully genotyped Diversity Outbred mice, revealing impacts of genetic variation on protein expression and lipid metabolism.

## Introduction

A foundational principle of cellular biology is that a cell’s genotype drives its phenotype. This link is mediated by the proteome, with variations in proteome status—often called the proteotype^1^—governing phenotypes of all kinds that manifest as cellular function and dysfunction. Thus, a cell’s proteotype encodes valuable insights into cellular function and clinical applications including disease diagnosis and progression monitoring. However, in order to extract these insights, approaches are needed to accurately and consistently profile the status of the proteome as well as account for variability at multiple levels including genetic diversity and environmental heterogeneity as well as technical variability. To overcome these hurdles, these approaches also need to contend with large numbers of samples.

To date, numerous targeted protein/pathway assays have been proposed for clinical uses, such as disease diagnosis and progression monitoring^2,3^. Targeted pathway proteomics aims to explore families of proteins that represent entire signaling pathways and gene categories with minimal sample input and instrument time but with maximum coverage and reproducibility. Commonly, the measurements are obtained through dedicated acquisition of tandem mass spectra (MS/MS) of peptides across a chromatographic gradient—sometimes called parallel reaction monitoring (PRM)^4,5^. Many dozens of peptides can be targeted in a single assay, and either retention-time scheduling or sequenced-matched, stable-isotope-labeled synthetic peptides are used as triggering events for the dedicated PRM and MS/MS scans^6,7^.

Sample multiplexing in proteomics through the use of isobaric tagging reagents (e.g., iTRAQ and TMT) is a powerful approach to increase the throughput and accuracy of protein quantification. Tandem mass tag (TMT) reagents allow for increased throughput as both 16-plex and 18-plex reagent sets are commercially available^8,9^. Previously, we combined sequenced-matched synthetic peptides as triggering events for TMT-labeled plexes^10,11^. However, this approach required a large investment in peptide synthesis and characterization such that assays cannot be created on the fly.

An instrument application programming interface (iAPI) is available for Orbitrap Tribrid mass spectrometers that permits the external computer to perform real-time analytics and trigger the collection of new scans on demand. A tribrid mass spectrometer is effectively multiple mass spectrometers combined, enabling the user to select multiple modes of fragmentation and to detect precursor and product ions using multiple mass analyzers that vary in speed, sensitivity, and resolution^12,13^. They thus have the potential to execute assays highly customized to each analyte. While these instruments usually run pre-defined methods, the iAPI enables Tribrid mass spectrometers to respond adaptively to the idiosyncrasies of each sample. Applications include real-time protein database searching with scan decisions made in milliseconds based on peptide sequence^14,15^ as well as adaptive decision-making in targeted proteomics assays^11^.

The Diversity Outbred (DO) mouse model is a powerful genetic resource to systemically evaluate how genetic variation influences protein expression and physiological phenotypes^16^. Moreover, genetic effects can be conserved between mouse and human homologs and be linked to human diseases^17,18^. This model is often not feasible for proteome-wide studies since many hundreds of animals often make up a cohort of DO mice to provide statistical power, and targeted proteomics could play an important role in analyzing large numbers of samples.

Here we present a next-generation targeted proteomics platform capable of utilizing TMT labeling with no need for synthetic peptide internal standards. The approach, termed GoDig, distills data from previously collected proteome-wide experiments into elution and spectral libraries to make real-time decisions via the iAPI during targeted assays. We removed the need for internal standard peptides so that nearly any previously observed peptide can be targeted, minimizing the effort needed for assay development. By performing real-time elution calibration, hundreds of target analytes can be multiplexed into single assays. We compiled the information needed to target any list derived from ~10 K human proteins and ~7 K mouse proteins as a resource accompanied by the GoDig assay builder website (https://wren.hms.harvard.edu/godig/) to simplify assay construction. We applied GoDig to measure protein abundances and investigated the impact of genetic variation on protein expression across 480 livers from the Diversity Outbred mouse model fed a high-fat diet. Each list of proteins was quantified across all 480 animals in <60 h, which is equivalent to 7.5 min of instrument time per sample. We identified previously unknown protein quantitative trait loci (pQTL). By interrogating a lipidomics dataset in these same animals^19^, pQTL were connected to lipid QTL and potential key modulators of lipid homeostasis were identified. In this way, we demonstrated that rapidly deployed targeted assays can measure the state of the proteome relating genetic variation to lipid phenotypes across a population of nearly 500 mice. GoDig has the potential to fundamentally transform targeted proteomics and serves as a cornerstone for the future development of a wide variety of pathway-specific measurement assays.

## Results

### GoDig assay design for measuring protein expression across entire pathways

**Fig. 1:**
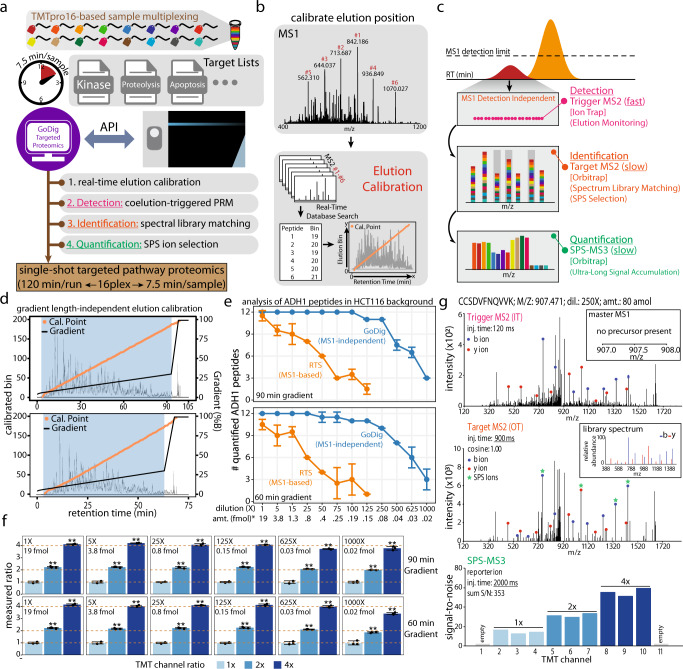
GoDig enables real-time profiling for targeted proteomics. GoDig uses real-time analytics to locate each target analyte relative to prominent background ions present in each sample **a**–**d**. The resulting quantitation is more sensitive than competing MS1-based approaches (**e**) and results in high-quality identification (**g**) and quantification (**f**) of each analyte. **a** GoDig governs several functionalities prompting a variety of scans to perform multiplexed targeted proteomics: (1) elution calibration in real-time using abundant MS1 peaks; (2) detection: coelution-triggered parallel reaction monitoring (PRM) scans for elution monitoring; (3) identification: high-resolution spectrum library matching; (4) quantification: synchronous precursor selection (SPS) and ultra-long injection MS3 collection. **b** Episodic elution position prediction. Periodically, a handful of MS2 spectra are taken to precisely determine position. **c** Once the elution window is calibrated, GoDig triggers fast PRM scans for lists of peptide targets in the window to monitor peptide elution without MS1 detection. If a target is detected, a high-resolution MS2 scan is collected for spectrum library matching and SPS ion selection, followed by MS3-based quantification. **d** Real-time elution calibration using ordered elution is robust to gradient changes. The orange dots represent the calibrated elution points, and the black line represents the LC gradient. **e** GoDig is appreciably more sensitive than MS1-based detection. Yeast ADH1 peptides (labeled with TMT at different ratios) were spiked into a TMT11-labeled HCT116 cell line background (0.5 µg on column). A total of 12 ADH1 peptides were targeted. Points represent mean ± s.d (*n* = 2 independent experiments). Source data are provided in the Source Data file. **f** The quantification results of selected dilutions. Bars represent mean ± s.d. (*n* = 3 independent measurements), **p ≤ 0.01 (two-sided Student’s *t*-test), *ADH1 on-column amount in the highest TMT channel. Dotted lines represent expected ratios. Source data are provided in the Source Data file. **g** An example of a GoDig inserted quantification event from the sample of 250X (80 amol) dilution. A trigger MS2 for CCSDVFNQVVK matched 16 fragment ions and triggered a high-resolution target MS2. Matched ions were cosine-correlated to the library spectrum (similarity = 1.00), and top fragment ions were selected for MS3 fragmentation and quantification. GoDig prompted the instrument to accumulate signal for 2000 ms for this quantification event.

To create each GoDig assay using the iAPI software for real-time analytics (Fig. 1a), we needed to target hundreds of peptides during an analysis without the use of internal standard peptides (ISPs). These ISPs provide two pieces of critical information for each peptide: (1) a precise retention time (RT), and (2) a reference peptide spectrum for identification^11,20^. RT scheduling can allow the multiplexing of target analytes into batches at the expected elution times, and the reference spectrum guards against false positive quantifications^21,22^.

To eliminate the need for making synthetic peptides and facilitate the use of any previously detected peptide as RT goalposts for better chromatographic resolution, we extended the idea that peptide elution orders remain relatively robust across runs regardless of liquid chromatograph (LC) variations^23^ and implemented a real-time elution calibration strategy within GoDig. Relative elution orders of all peptides from deeply fractionated datasets are binned to certain RT intervals (e.g., 30 s) to construct an elution library. Meanwhile, all peptide spectral matches are converted into a spectral library for online spectral matching (identification). Thus, using GoDig, in a targeted single-shot experiment, the most intense peaks from an MS1 scan are selected only periodically, and subsequent MS2 scans are collected and searched using a real-time database search algorithm (Comet)^14^. The elution information for the resulting peptide IDs is extracted from the elution library to calibrate the elution in real-time (Fig. 1b; Supplementary Fig. 1). It is worth noting this strategy is highly robust to different LC gradients (Supplementary Fig. 2a, b, j, k), sample types (Supplementary Fig. 2a, b) and even swapping of isobaric labeling reagents (i.e., 11- or 16-plex TMT reagents) (Supplementary Fig. 2c, d). In addition, elution order was found to be stable across different LC stationary phases (Supplementary Fig. 2f–i).

Once the current elution point is calibrated, all peptide targets predicted to elute within a specified window are monitored continuously via rapid PRM scans in the ion trap mass analyzer (IT-PRMs). GoDig has real-time access to each IT-PRM and upon successful matching of ≥6 fragment ions, it prompts a high-resolution MS2 scan in the Orbitrap (OT-MS2). The resulting OT-MS2 is matched using cosine similarity scoring to the library spectrum^24,25^. Fragment ions are also examined for purity (no interfering peaks) and then selected for a synchronous precursor selection (SPS) MS3 scan^26^ to retrieve quantitative information with minimal interference (Fig. 1c, g). Because we have high certainty of the identification and elution position, the inserted SPS-MS3 scans can have ultra-long ion accumulation times (up to 2000 milliseconds) to dramatically increase sensitivity. We also note the spectra from TMT11- and TMTpro16-labeled peptides are highly comparable, making it possible to transfer libraries among experiments using either reagent (Supplementary Fig. 2e).

### Validating the GoDig method with spike-in yeast alcohol dehydrogenase 1 (ADH1) protein

To validate GoDig, we labeled and combined tryptic peptides derived from a yeast protein standard (ADH1) to have final ratios of 1, 2 or 4 in triplicate using the middle 9 channels of the TMT11 reagents. To mimic a highly complex background, we labeled tryptic peptides from whole human HCT116 lysate at a 1:1 ratio across the 11 channels. ADH1 peptides were then serially diluted at known concentrations and spiked into 0.5 µg of HCT116 prior to LC-MS-GoDig analysis. The on-column amount was defined by the absolute amount (in fmols) of ADH1 in the highest TMT channel (ratio of 4), and the highest dilution (1X) contained 19 fmol in that channel. Elution and spectral libraries were built using data from duplicate injections of 1X ADH1 peptides in 0.5 µg HCT116 backgrounds using a 90-min gradient. Twelve ADH1 peptides were identified and subsequently targeted in serial dilutions of up to 1000X (20 attomoles on-column), and the same 90-min gradient or a shorter 60-min gradient was used. For comparison, we also acquired data using the MS1-based detection with real-time search (RTS)-enabled shotgun proteomics approach as a reference^14,27^. Regardless of the gradient used, GoDig successfully calibrated elution profiles on the fly (Fig. 1d), showing the robustness of the strategy to different gradients. Because GoDig is not dependent on MS1 detection, ADH1 was quantified by multiple peptides throughout the dilution series (Fig. 1e). Even at the 1000X dilution with only 20 attomoles of ADH1 on-column, GoDig successfully quantified 3 peptides (Fig. 1e), achieving good accuracy and statistical power to differentiate triplicates of different ADH1 amounts (Fig. 1f). These data demonstrate that despite the precursors of targets being far below the general detection limit for MS1-based triggering, GoDig still quantified the targets of interest. To discern subtle changes and reduce interference, GoDig allowed up to 2000 ms ion accumulation time for very weak signals, as exemplified by the ADH1 peptide, CCSDVFNQVVK, with 80 attomoles on column (Fig. 1g).

### Benchmarking GoDig against deeply fractionated datasets from four human cell lines

**Fig. 2:**
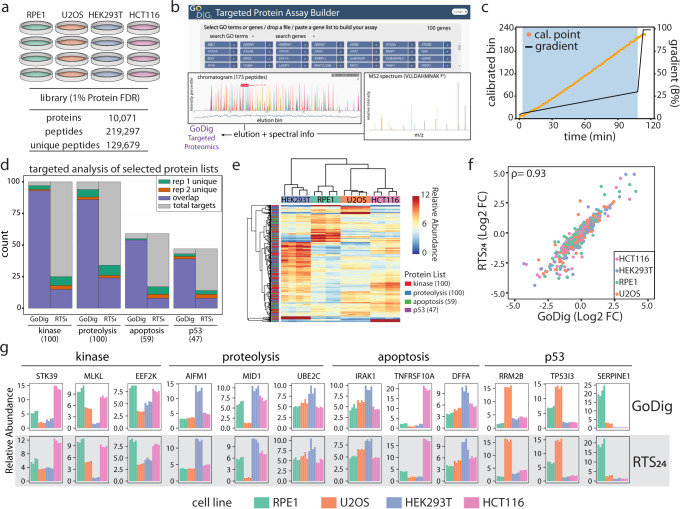
Accurate and complete profiling of protein families via GoDig. Overview: To demonstrate that GoDig may be used to quantify arbitrary protein families with high accuracy and coverage, we targeted proteins from four representative families for quantification across human cell lines (**a**–**c**). High coverage was achieved across all four families (**d**) and significant variations in abundance were observed across cell lines for each protein family (**e**). Quantitative profiles were consistent between methodologies (**f**, **g**), with GoDig performance equivalent to, if not better than, that achieved via RTS with fractionation. **a** Biological quadruplicates of RPE1, U2OS, HEK293T, and HCT116 were processed and labeled with TMTpro16. To construct the libraries for elution prediction and spectral matching, 24 fractions were generated, and each was analyzed with a 2-h gradient. The library consisted of more than 10,000 targetable proteins. **b** GoDig targeted protein assay website (http://caribou.med.harvard.edu/godig/) was used to construct different protein assays. Input can be either gene symbols or entire pathways (e.g., GO terms, Reactome). **c** Real-time elution calibration in a 2-h single-shot GoDig experiment using the elution library constructed across 24 fractions. **d** GoDig experiments targeting four separate gene lists were collected. Up to 100 members were selected from each category [gene lists obtained from MSigDB^31, 58^]. Two technical replicates were collected using GoDig or MS1-detection-based RTS_1_, and high reproducibility was achieved. GoDig quantified more than 94% of the proteins on each list. Source data are provided in the Source Data file. **e** Hierarchical clustering of all proteins quantified by GoDig from four targeted lists. **f** Correlation between GoDig and fractionated RTS (RTS_24_) protein quantification. The fold change represents the ratio between the mean of a single cell line and the mean of the other 3 cell lines. The Spearman correlation is 0.93. Source data are provided in the Source Data file. **g** Example bar charts for protein quantifications using either GoDig (2-h analysis) or RTS_24_ (48-h analysis). Source data are provided in the Source Data file.

After the initial validation of GoDig using spike-in ADH1 protein, we generated tryptic peptides from biological quadruplicates of four human cell lines—RPE1, U2OS, HEK293T and HCT116 (Fig. 2a). Peptides were labeled with TMTpro16 reagents. We first created a library using an unfractionated sample (Lib_1_) and analyzed it in duplicate to construct libraries. Without sample fractionation, detected peptides are abundant species in Lib_1_ and their elution variances are only minimal. Therefore, we were able to target 2600 peptides in a single GoDig experiment (Supplementary Fig. 3b, c; Supplementary Data 6), achieving comparable target multiplexing capacity as two previous targeted strategies^5,28^ while dramatically increasing sample multiplexing (16x) and quantification accuracy by incorporating TMT-based isobaric labeling^9,29^ and the SPS-MS3 technology^26^. However, similar to the previous reports^5,28^, unfractionated samples, and the resulting Lib_1_ covered only the abundant fraction (top 20–30%) of the whole proteome (Supplementary Fig. 3d, e, f). The rest of the proteome remained inaccessible due to the massive expression dynamic range, spanning several orders of magnitude.

As our goal was to target proteins at all abundances, we benchmarked its utility by constructing libraries from a deeply fractionated sample. 24 fractions of the same TMTpro16-labeled 4 cell line sample were collected using offline basic pH reversed-phase chromatography^30^. Each fraction was analyzed using a 2-h method. Data from 24 fractions were searched and filtered to a protein false discovery rate of 1%. We identified 129,679 peptides from 10,071 expressed proteins (~13 peptides/protein; Supplementary Data 7) for inclusion in the deep library (Lib_24_) (Fig. 2a). Using the web-based GoDig assay builder (Fig. 2b; Supplementary Fig. 9), we built assays in silico for four selected MSigDB protein categories^31^, namely protein kinases, proteolysis, apoptosis and p53 signaling, allowing initially up to 100 protein targets in each category using 3 peptides (if available) for each.

To calibrate the elution window, periodically (every 15 s), the top 6 peaks in an MS1 scan were fragmented, identified via real-time database search (RTS), and assigned precise elution positions. Compressing 24 fractions into a single shot, GoDig still presented robust elution calibration (Fig. 2c; Supplementary Fig. 2j, k). We initially elected to use wider elution windows (~3.5 min) than the experiments targeting 2600 peptides using Lib_1_ (~0.5 min) for targeting each peptide in Lib_24_ to account for elution variation across the 24 fractions (Supplementary Fig. 2j, k) used for the libraries, and we allowed ultra-long ion accumulation (up to 2000 ms) to assure successful and accurate quantification.

To evaluate the sensitivity and reproducibility of GoDig when targeting proteins in the Lib_24_, we selected four categories of proteins and two technical replicates for each category were collected using either GoDig or RTS analyses of the unfractionated sample (RTS_1_). On average, 94% of targets were quantified across the four categories using GoDig, as opposed to just 29% by RTS_1_ (Fig. 2d). Despite the significant improvement in sensitivity, the two technical replicates using GoDig had 88% of the proteins quantified reproducibly on average (Fig. 2d), whereas the two RTS_1_ technical replicates only commonly quantified 17% of the targets, highlighting the notably better reproducibility by GoDig. Comparing quantification of the single-shot data collected by GoDig to the RTS data collected with 24 fractions (RTS_24_; Supplementary Data 1) revealed a strong correlation with a Spearman *ρ* = 0.93 (Fig. 2f, g), even though GoDig needed only 1 µg total peptide and a 2-h single-shot run for each 16-plex experiment, whereas RTS_24_ consumed 100 µg total peptide for fractionation and each of the 24 fractions required a 2-h analysis. Hierarchical clustering with the GoDig-quantified proteins showed clear separation among cell types as expected (Fig. 2e).

**Fig. 3:**
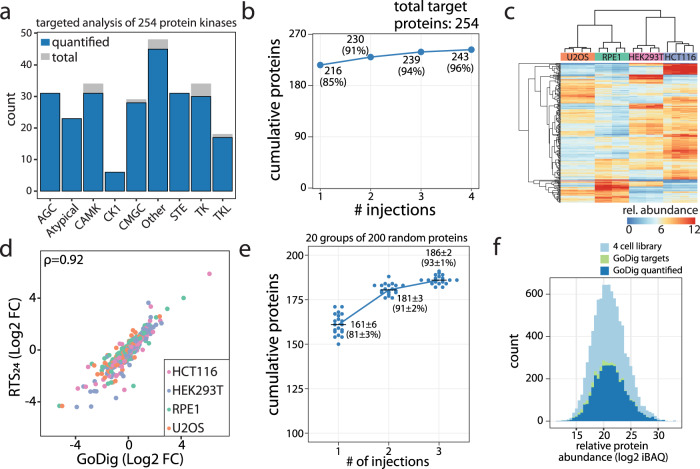
Evaluating the upper limit for proteome-wide targeting with lists of ~200 proteins. Low abundance proteins are much more difficult to quantify, and they require ultra-long accumulation times. Using lists of 200 or more proteins is possible, but injection times are limiting across the full dynamic range of the proteome. **a**–**d** 254 protein kinases (479 peptides) were selected from the Lib_24_ (~10,000 proteins) built with the TMTpro16-labeled four human cell sample as targets, and four consecutive GoDig runs were performed using ~1 µg unfractionated sample while enabling the close-out feature. Close-out removes peptides/proteins from the list once they have been quantified. In total, 216 kinases were quantified in a single GoDig experiment, and 96% of all targeted kinases were quantified using several injections with close-out. **a** Kinase subtypes targeted and quantified. Source data are provided in the Source Data file. **b** Protein quantification events in the kinome assay including multiple GoDig injections with close-out. Source data are provided in the Source Data file. **c** Hierarchical clustering shows good separation by cell type. **d** GoDig generated result in good agreement with the result acquired by analyzing 24 fractions of the sample with RTS (RTS_24_) (Spearman’s *ρ* = 0.92). The fold change (FC) is calculated as the ratio of the mean of one cell line compared to the mean of the other three cell lines. Source data are provided in the Source Data file. **e**, **f** Twenty gene lists were selected from the 10,000-protein library. Each list contained 200 randomly selected targets, each with up to three peptides if available. In total, 4000 proteins (20 × 200 member lists) were targeted. **e** Three consecutive injections were performed with the close-out function enabled to assess coverage. On average 93% of all targets were successfully quantified (Source data are provided in the Source Data file), and **f** their natural abundance spanned full dynamic range of the proteome sample.

Our larger biological goal is to target entire families and pathways of proteins, which necessitates an evaluation of the number of proteins that can be targeted in a single assay. The number of proteins that can be targeted is limited by the ultra-long signal accumulation times allowed for each individual target to assure proteome-wide sensitivity and accuracy as well as how many targets are concurrently eluting at a certain time point. Having targeted up to 100 proteins (~300 peptides) with widely varying native abundances across the four categories studied, we next sought to evaluate the use of larger lists of proteins. We performed two experiments. Since protein kinases are a popular protein class for expression profiling, we targeted 254 protein kinases using GoDig (Fig. 3a). With a single run, GoDig quantified 216 targets in 2 h, and the total number of quantified kinases increased to 243 (96%) after adding data from four consecutive injections using a close-out functionality to increase sensitivity (Fig. 3b).

As a more representative benchmark, we generated 20 separate target lists, each containing 200 random proteins with distinct endogenous abundance levels derived from more than 10,000 expressed proteins (Fig. 3f). We allowed up to three peptides to be used for quantification of each target and collected three consecutive runs for each list closing out previously quantified proteins (See Methods). In all, 81% of the list for single injection experiments were detected and quantified. Over 93% of all targeted proteins were successfully quantified after three injections (Fig. 3e), and their endogenous expression levels spanned the full dynamic range of the proteome as suggested by the estimation of their absolute abundances (iBAQ values)^32,33^ (Fig. 3f). We observed <10% CV among biological replicates (Supplementary Fig. 4a) and a good correlation (Spearman *ρ* = 0.91) to the RTS data collected with 24 fractions (Supplementary Fig. 4c). These data demonstrate smaller lists (up to 100 members) allow for deeper list coverage at full proteome depth. While 200-member lists are possible, modifications such as the limiting the number of peptides per protein are necessary to achieve >90% list coverage.

### Investigating the consequences of genetic variation on protein expression in Diversity Outbred (DO) mouse livers

Powerful mouse models are available to biologists to study the genotype-phenotype relationship often including hundreds of animals with full genotyping and measured phenotypes^16,34^. For example, Chick and coworkers performed a large-scale untargeted proteomics study and mapped protein quantitative trait loci (pQTL) using 192 DO mouse livers^35^, and Linke and coworkers also mapped thousands of lipid QTL in liver using DO mice^19^. We were motivated to build upon these works to fill in the gaps for certain protein families that are especially relevant to lipid homeostasis biology as the mice were fed a high-fat diet. We first performed a pilot study exploring the full proteome expression of the 8 founder strains (*n* = 8 for each founder; four males and four females) from which the DO cohort was derived. Approximately 75% of all quantified liver proteins (5566 out of 7397) had significant differences in at least one strain relative to the C57BL/6J mouse (Supplementary Fig. 5; Supplementary Data 2). Such expression differences in founders support the presence of regulatory genetic variants (pQTL). We found it attractive to leverage the founder strain resource, the existing pQTL liver dataset^35^, and the existing lipid QTL dataset^19^ to measure pathway-wide how genetic variation affects lipid homeostasis.

**Fig. 4:**
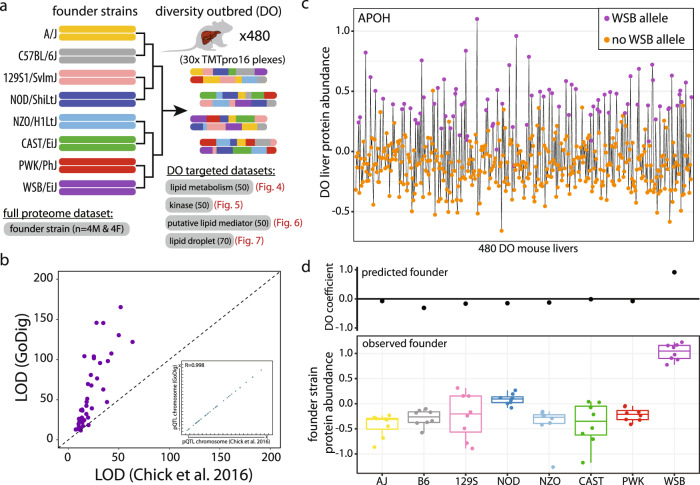
Targeted pQTL analyses of livers from 480 genotyped DO mice fed a high fat diet. **a** DO mouse livers were obtained from the Keller et al. study^36^ and processed into thirty TMTpro16plex experiments. For GoDig analyses, four targeted gene lists included lipid metabolism-related proteins, kinases, putative lipid metabolism mediators and lipid droplet proteins were generated. In addition, livers from 64 animals from the 8 DO founder strains (*n* = 8 animals for each strain) were processed and full proteome profiling was obtained using a real-time search (RTS)-based shotgun approach^14^. **b** The new LOD scores are higher using GoDig across 480 mice than the scores generated using 192 mice from Chick et al.^35^. The inset shows the great pQTL positional correlation between the re-analysis using GoDig and the result by Chick et al.^35^. Source data are provided in the Source Data file. **c** Example of APOH protein expression measured by GoDig across the full dataset (*n* = 480 animals). DO mice are arranged by TMTpro16 labeling order. Mice with ≥1 predicted allele from WSB are colored purple and have higher standardized APOH expression. Source data are provided in the Source Data file. **d** GoDig identified a local pQTL for APOH on chromosome 2. The founder allele effects from the pQTL in the DO population is highly correlated with the measured abundance in the eight founder strains (*n* = 8 animals for each strain). Bottom border, interior line, and top border in the box plot represent the 1st quartile, median, and 3rd quartile, respectively. Source data are provided in the Source Data file.

Using GoDig, we focused on specific subsets of proteins across 480 livers from DO mice fed a Western-style diet high in fat and sucrose (Fig. 4a**;** Supplementary Fig. 6)^36^. Peptides from the 480 livers were randomly assigned to 30 TMT groups and labeled with the TMTpro16 reagents. We first tested GoDig by targeting 50 lipid metabolism-related proteins, among which 49 already had known pQTL. The 50 target proteins were quantified by GoDig using a 2-h GoDig method, and all 480 mice were surveyed over 60 h (Supplementary Data 3). All targets achieved 100% data completeness. Most known pQTL (44 out of 49 [90%]) were recapitulated, and we observed remarkably increased log odds ratio (LOD) scores reflecting increased statistical power (Fig. 4b). Protein apolipoprotein H (APOH) exemplified a local pQTL where a genetic variation in the APOH locus on chromosome 11 governed the protein’s expression. Genetic mapping indicated that DO mice with allelic contribution from the WSB founder strain presented elevated levels of APOH (Fig. 4c). The predicted allelic contribution at the pQTL agreed with the expression profile in the founder strain data (Fig. 4d). In addition to recapitulating known pQTL, seven new pQTL were also identified, including five distant and two local pQTL (Supplementary Data 4).

**Fig. 5:**
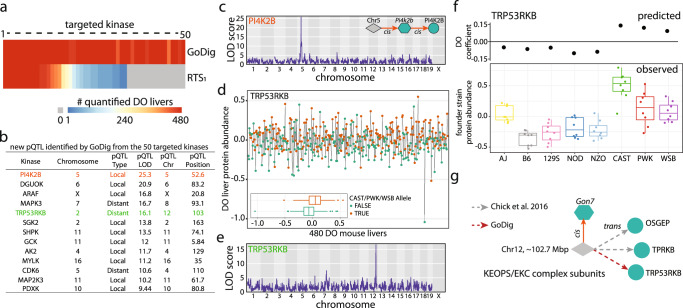
GoDig identifies previously undetected pQTL across 50 targeted kinases. Experimental overview is illustrated in Fig. 4a. Fifty kinases, including forty-six that had no annotated pQTL in Chick et al.^35^, were targeted and measured with GoDig. **a** Heatmap of kinase detection across the 480 samples. Data were collected using GoDig or single-shot RTS (RTS_1_) and a 2-h method/plex. Gray indicates the protein was not quantified in any sample. Less than 30% of kinases were consistently detected across the 480 samples using MS1-based detection (RTS_1_). **b** New pQTL identified by GoDig for the 50 targeted kinases. **c** Genome scan for PI4K2B pQTL. LOD score represents the statistical strength of genetic association. PI4K2B abundance is regulated by a local pQTL on chromosome 5. PI4K2B abundance is likely regulated through a transcriptional mechanism because a corresponding local eQTL was reported by Chick et al.^35^. The founder allele effects of the eQTL is consistent with that of the pQTL identified by GoDig (Supplementary Fig. 7). **d** TRP53RKB protein expression across the 480 DO mice are arranged along the *x* axis by TMTpro16 labeling order. The inset shows the boxplot of TRP53RKB protein abundance grouped by the predicted presence of alleles from CAST, PWK or WSB, which are expected to have higher TRP53RKB expression. Bottom border, interior line, and top border in the box plot represent the 1st quartile, median, and 3rd quartile, respectively. Source data are provided in the Source Data file. **e** Genome scan for TRP53RKB. TRP53RKB abundance is regulated by a distant pQTL on chromosome 12. **f** Predicted founder allele effects for TRP53RKB kinase across the DO population is highly correlated with the observed founder protein abundance (*n* = 8 animals for each strain). Bottom border, interior line, and top border in the box plot represent the 1st quartile, median, and 3rd quartile, respectively. Source data are provided in the Source Data file. **g** The abundance of KEOPS complex subunits OSGEP, TRPKB and TRP53RKB are regulated by a distant pQTL on chromosome 12 around 102.7 Mbp. The transcript abundance of the core complex member, *Gon7*, is regulated locally by the same locus, consistent with *Gon7* being the core regulator of the three other subunits.

We next sought to identify pQTL that were missed in the previous study^35^ but might be measured by targeted approaches efficiently across a larger DO mouse cohort to more completely account for the genetic variation and phenotypic diversity. A GoDig assay was built to target 50 kinases without previously annotated pQTL, yet each of the 50 kinases presented notable changes in the founder strain proteome-wide resource (Supplementary Data 2), suggesting that pQTL were present. GoDig achieved an average of >99% data completeness. As a comparison, we ran the same samples using a real-time search (RTS) method with the identical gradient. The average data completeness with RTS was only 38%, and 16 of the targets were never quantified in any sample (Fig. 5a). We identified 13 pQTL that eluded previous identification. For example, PI4K2B abundance was regulated by a local pQTL on chromosome 5, and the effect was likely imposed through a transcriptional mechanism because a corresponding local eQTL has been reported^35^. The pQTL mapping suggested alleles from three founder strains (CAST, PWK and WSB) harbor genetic variants that cause higher PI4K2B expression (Fig. 5b, c; Supplementary Fig. 7). Besides local genetic effects, we observed several distant pQTL, including one for TRP53RKB (Fig. 5b, d). TRP53RKB is a member of the EKC/KEOPS complex, and the distant pQTL resided on chromosome 12 near 102.7 Mbp (Fig. 5e). Allelic signatures predicted positive contributions from founder strains CAST, PWK and WSB and agreed with the measured founder strain abundance (Fig. 5d, f). Upon closer investigation, we found that the *Gon7* gene, which encodes GON7 as a subunit of the complex, is within the pQTL locus, and two other complex subunits, OSGEP and TPRKB, also have distant pQTL pointing to the same locus. An eQTL with allele effects matching the distant pQTL was observed for the *Gon7* transcript (Fig. 5g). Therefore, we infer that the EKC/KEOPS complex subunits are modulated by local genetic variants in *Gon7* in the liver.

### Identification of putative protein modulators for hepatic lipid metabolism

**Fig. 6:**
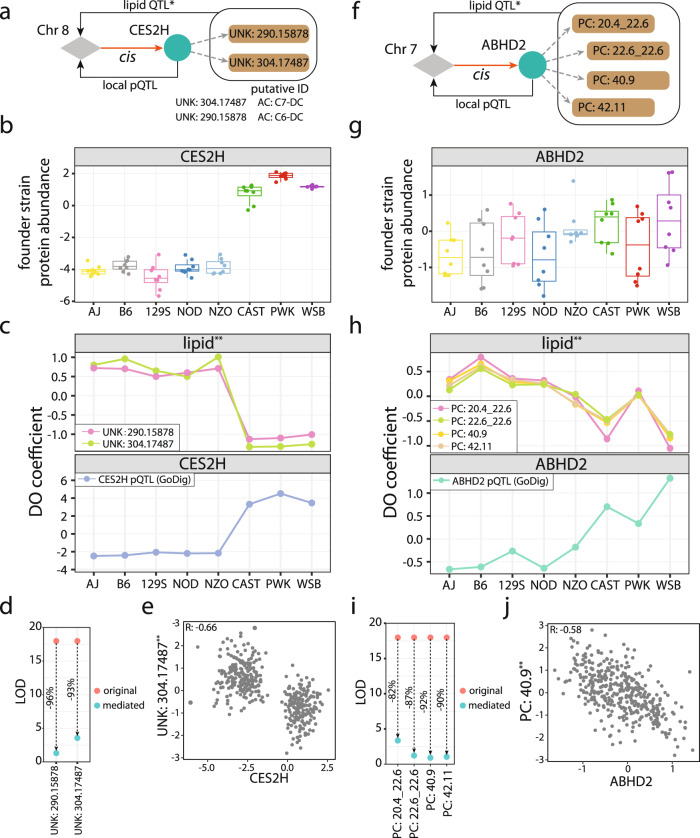
Assigning drivers to previously reported lipid QTL detected in DO mouse livers by Linke et al.^19^. **a** Example of two lipid QTL that map to the same locus on chromosome 8. GoDig analysis identified a local pQTL regulating the abundance of CES2H and the abundances of two lipids with unknown identities. Based on functional annotation of CES2H and its homologs and mass spectra of the two unknown lipids, the two lipid species are putatively identified as acylcarnitine species with molecular formulas C_14_H_26_NO_6_ (e.g., pimeloylcarnitine) and C_13_H_24_NO_6_ (e.g., methylglutarylcarnitine) (Supplementary Fig. 8). **b** Liver protein abundance of CES2H in the 8 founder strains (*n* = 8 animals for each strain). Bottom border, interior line, and top border in the box plot represent the 1st quartile, median, and 3rd quartile, respectively. Source data are provided in the Source Data file. **c** Founder allele effects observed at the QTL for the two unknown lipids and local pQTL for CES2H. The lipid QTL have allele effects that are negatively correlated with that of CES2H (mean Pearson R: −0.98). Source data are provided in the Source Data file. **d** Mediation analysis of the two unknown lipids’ levels through CES2H results in ~95% LOD score reductions. **e** CES2H abundance in DO livers is negatively correlated with UNK: 304.17487 (*R* = −0.66). Source data are provided in the Source Data file. **f** A cis-pQTL on chromosome 7 regulates ABHD2 abundance and ABHD2 further regulates abundances of four phosphatidylcholines (PCs). **g** Protein abundance of ABHD2 in eight founder strains (*n* = 8 animals for each strain). Bottom border, interior line, and top border in the box plot represent the 1st quartile, median, and 3rd quartile, respectively. Source data are provided in the Source Data file. **h** Founder allele effects estimated at the QTL for 4 PCs and local pQTL for ABHD2. The lipid QTL have allele effects negatively correlated to that of ABHD2 (mean Pearson *R*: −0.95). Source data are provided in the Source Data file. **i** Mediation of 4 PC lipid levels through ABHD2 reveals ~90% LOD score reductions. **j** ABHD2 abundance in DO livers is negatively correlated with PC:40.9 (*R* = −0.58). *indicates quantitative values obtained from Linke et al.^19^. Source data are provided in the Source Data file.

Proteins are essential functional units linking genotype to phenotype. A recent lipidomics study by Linke and coworkers reported thousands of liver lipid QTL with unknown gene and protein drivers^19^. To bridge the gap between genetic variants and lipid modulation, we selected 50 proteins that were within some of these liver lipid QTL loci and had founder strain abundances similar to the allele effects of the lipid QTL. After GoDig analysis, we identified 41 pQTL (Supplementary Data 4). Two examples of putative lipid modulators are CES2H (Fig. 6a) and ABHD2 (Fig. 6f). Two lipids with unknown identities, labeled previously as UNK:304.17487 and UNK: 290.15878, had QTL mapped to chromosome 8 in the region of the gene, *Ces2h*. CES2H abundance in the founder strains was negatively correlated with the inferred allele effects of the lipids (Fig. 6b, c). A local pQTL for CES2H was also detected using GoDig, with matching allele effects (Fig. 6c). We included CES2H abundance as a covariate in the lipid QTL model and examined how each QTL LOD score changed (Supplementary Data 5). If a protein’s levels were co-regulated with a lipid’s abundance, then regressing out its abundance during QTL scanning should result in a reduced LOD score. The inclusion of CES2H abundance caused ~95% reduction of each lipid’s LOD score, and the near-complete reduction suggested a primary regulatory role for CES2H. Correspondingly, CES2H abundance was negatively correlated with each lipid’s abundance (Pearson *R* = −0.66 with UNK:304.17487 and −0.55 with UNK:290.15878) (Fig. 6e; Supplementary Fig. 8c).

We next used the gathered genotypic, proteotypic and phenotypic information to assist in the identification of the unknown lipids. CES2H belongs to a protein family of 8 mouse carboxylesterases. Although the specific function of CES2H has not been fully elucidated, the family and its human homolog are known to possess acylcarnitine (AC) hydrolase activity^37,38^. We extracted MS2 scans for the lipids from the lipidomics data, and characteristic ions of acylcarnitine at *m/z* 60.08 (trimethylamine), 85.03 (C_4_H_5_O_2_^+^), the neutral losses of 59 and 161 Da corresponding to the loss of the trimethylamine moiety, and the loss of the carnitine backbone were present in the scans (Supplementary Fig. 8a, b)^39,40^. Therefore, we identified the two unknown lipids as putative AC species with ion formulas C_14_H_26_NO_6_^+^ (e.g., pimelylcarnitine) and C_13_H_24_NO_6_^+^ (e.g., methylglutarylcarnitine). Similarly, we found a local pQTL for ABHD2 on chromosome 7, within the same locus as the QTL for four phosphatidylcholines (PCs). ABHD2 abundance in founder strains and its inferred allele effects from the 480 DO mouse livers negatively correlated with those of the four PCs (Fig. 6g, h). Correspondingly, ABHD2 abundance negatively correlated with the PCs’ abundances across 480 DO livers (Fig. 6j and Supplementary Fig. 8d–f. Mediating PCs’ abundances through ABHD2 led to LOD reductions ranging from 82 to 90% (Fig. 6i), consistent with ABHD2 inhibiting PCs.

### Identifying a potential modulator for hepatic lipid storage

**Fig. 7:**
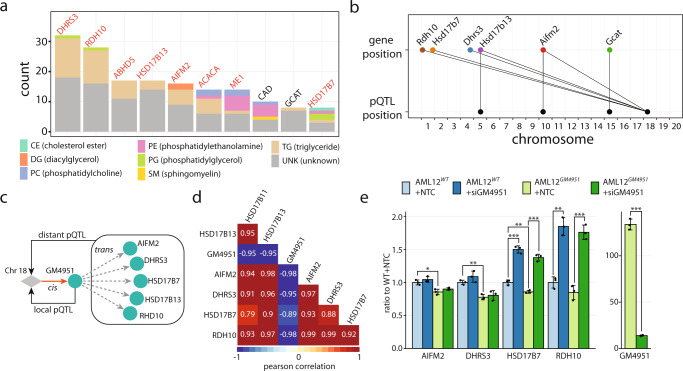
Mediation of lipid QTL reveals a key regulator for lipid droplets. The four GoDig analyses targeting 220 proteins across 480 mouse livers were combined. Each protein was evaluated as a potential mediator of QTL for 2,269 lipids in the livers from Linke et al. Mediation results were filtered using an LOD score reduction >50% and z-score < −4. Resulting protein-lipid pairs were considered as being co-regulated. **a** Bar chart representing the number of lipids from Linke et al. with which each protein is co-regulated. The top 10 proteins are shown. Proteins known to have lipid droplet localization are in red text. Lipids are colored based on categories. **b** Gene and pQTL positions of the top 6 proteins with the most co-regulated lipids and identified pQTL. Five proteins (AIFM2, DHRS3, HSD17B7, HSD17B13 and RHD10) have a distant pQTL mapping to a hotspot on chromosome 18 at 60 Mbp. **c** The *Gm4951* gene is within the hotspot on chromosome 18 and is a potential regulatory protein for lipid droplet homeostasis. A cis-pQTL for liver GM4951 is consistent with it being the key regulator for the abundance of 5 lipid droplet-related proteins that have distant pQTL at the same locus. **d** Allele effects of the distant pQTL of AIFM2, DHRS3, HSD17B7, HSD17B11, HSD17B13 and RHD10 are positively correlated among themselves (mean Pearson *R* = 0.93), whereas they are negatively correlated with that of the local pQTL of GM4951 (mean Pearson *R* = −0.95). **e** Protein abundance m**e**asurements in AML12^*WT*^ and AML12^*GM4951*^, treated with non-targeting control (NTC) or siGM4951 (*n* = 3 biological replicates). Two sided Student’s *t-*tests were performed. p*<0.05, **p < 0.01, ***p < 0.001. Source data are provided in the Source Data file.

To further explore lipid homeostasis biology, we sought to identify drivers of lipid droplet formation in liver by integrating genotype, proteotype and phenotype. Thus, we quantified using GoDig 70 proteins annotated to the lipid droplet proteome^41^. In total, 31 pQTL were identified with a *q*-value < 0.1, including 17 that were not annotated by Chick and colleagues^35^. We hypothesized that lipids primarily localized in lipid droplets would covary with proteins having a similar compartmental distribution. We included all 220 proteins quantified so far by GoDig and treated each one as a covariate to perform mediation analysis for each of the reported 2269 lipid QTL^19^. Proteins were ranked according to the number of lipids with which they were co-regulated (LOD reduction>50% and Z-score < −4). Notably, 8 out of the top 10 proteins with the most co-regulated lipid QTL had known lipid droplet localization (Fig. 7a; Supplementary Data 5)^41^. Most of the covarying lipids with known identities are triglycerides (Fig. 7a), consistent with them being the major component in lipid droplets^42^. We also noted that 5 of the top 10 proteins had distant pQTL mapping to a hotspot on chromosome 18 at 60 Mbp (Fig. 7b), indicating the existence of a key lipid droplet driver within this locus. A recent study by Schwerbel et al.^43^, proposed GM4951 (a.k.a IFGGA2) as a regulator of hepatic lipid storage, and the *Gm4951* gene is within the tightly linked cluster of pQTL on chromosome 18. GM4951 displays a lysosomal/endosomal profile when mice are fed a low fat diet but redistributes primarily to lipid droplets when fed a high fat diet^43^. We were able to identify a local pQTL for GM4951. While the allele effects of the distant pQTL of RDH10, HSD17B7, DHRS3, HSD17B13 and AIFM2 were positively correlated among themselves (average Pearson *R* = 0.93) because of coregulation, the average Pearson correlation between those and the local pQTL of GM4951 was −0.95 (Fig. 7d). This finding points to a conclusion that GM4951 is potentially a key suppressor of lipid accumulation and has a preferential lipid droplet localization with a high fat diet (Fig. 7c).

Following the identification of GM4951 as a probable regulator of the five other protein’s levels, we overexpressed it in mouse hepatocyte AML12 cells (AML12^*GM4951*^) to validate the finding and investigate its biological implications. Knockdown of GM4951 with siRNA was introduced as another perturbation. Each treatment group was generated in biological triplicate (AML12^*WT*^ ± siGM4951, AML12^*GM4951*^ ± siGM4951), and their proteomes were analyzed using TMTpro16 and RTS technology^14^. RDH10, HSD17B7, DHRS3, and AIFM2 were quantified in the experiment. Overexpressing GM4951 led to downregulation of the four proteins, whereas siGM4951 treatment caused enhanced expression of the four proteins (Fig. 7e). The observed co-regulation along with the identification of the distant pQTL confirmed that GM4951 is responsible for regulating the four protein abundances.

## Discussion

Rapidly quantifying (sub)proteomes will be essential to linking genotype to phenotype in experimental systems that realistically account for biological diversity. Though targeted assays have been useful in this regard, they have been painstaking to develop. Through real-time analytics, much of the up-front set-up cost has been obviated, facilitating rapid development and deployment of these assays. Moreover, by incorporating elution-order scheduling and maximizing gas-phase enrichment, these assays have been made practical across the breadth of the detectable proteome. Finally, by incorporating TMT multiplexing, we have enhanced throughput, boosted sensitivity (through pooling of MS1 precursor and MS2 fragment signal), minimized missing values, and enabled more complicated experimental designs. All of this serves to frame a more accurate picture of the proteome and completely quantify proteome state and relate that state back to genotypic variation and forward to phenotypic variability.

Here we aim to demonstrate using GoDig to more fully explore proteotypic variability across large cohort of biological samples. Using GoDig, peptides from proteins representing entire pathways or gene categories that have been identified previously can be targeted using real-time analytics. Only two pieces of information are required: (i) the predicted elution position and (ii) an already-acquired fragmentation (MS/MS) spectrum for identification. We found the assay’s absolute sensitivity to be in the low attomole range (Fig. 1e) due to ultra-long ion injection times for the MS3 scans (up to 2000 milliseconds). In this way, a proteome-wide experiment as in Fig. 2 where 10,000 proteins (from 130,000 peptides) comparing expression across 4 cell lines is collected first and then used as a library. Next, lists of 50–200 members derived from any grouping of those 10,000 proteins can be targeted using multiple peptides per protein, and the generation of corresponding proteins assays is remarkably simplified with the GoDig target protein assay builder (Fig. 2b; Supplementary Fig. 9). Across the pathways and categories examined in this study, we found that more than 94% of targeted proteins were quantified, and their expression mirrored what was found with full proteome-wide datasets (Fig. 2d, f, g). We note the peptide library is expandable and future datasets collected with samples derived from different specimens will be continuously merged to the existing library toward global proteome coverage (Supplementary Fig. 10).

Previously, ordered elution has been proposed as a potential avenue to avoid retention time scheduling and to boost list size for targeting^5,23,28^. By combining this idea with real-time analytics, GoDig predicts in real time which peptides are most likely to be eluting at a given elution position and then inserts monitoring PRM scans via the API for just that subset. In this way, hundreds of proteins from GO categories or entire pathways can be targeted (Fig. 2c). The largest list of peptides attempted in this work contained 2600 peptides from ~900 relatively abundant proteins (Supplementary Fig. 3; Supplementary Data 6), and over 98% of them were quantified in a single GoDig experiment with ~3% CV across technical replicates (Supplementary Fig. 3b). However, to allow depth rather than just breadth, 2000 ms ion accumulation times are routinely used when querying proteins with abundance spanning the dynamic range of the entire proteome. In that regard, the largest list of proteins attempted at full-proteome depth in this work contained 254 protein kinases (479 peptides) (Fig. 3a–d). GoDig quantified 216 from a single injection, and 243 from total 4 consecutive injections. Using 20 random lists of up to 200 proteins of all abundances also resulted in >93% dataset completion. Since up to 2000 ms are consumed to quantify each peptide (using an MS3 scan with an ultra-long injection), the upper limit for the assay as described or with slight changes would be between 100 and 200 proteins in a single 2-h analysis (Fig. 3e).

Sample multiplexing has many salient advantages including (i) essentially no missing values within a single plex—all 16 measurements are collected simultaneously for each peptide, (ii) accurate quantification based on stable isotope dilution theory, (iii) the ability to accommodate complex experimental designs within one plex including replicates, positive and negative controls, dose-response, and time series data, and (iv) up to 16-fold higher throughput. The founder dataset is an excellent example of these advantages. Fully utilizing the 16-plex reagents allowed livers from all eight founder strains with one male and one female to be included in a single plex. In this way just four 16-plexes contained the entire experiment (64 livers) and all relevant comparisons could be made within a single plex (Supplementary Fig. 5).

Our DO mouse study included liver samples from 480 mice that were randomized into 30 separate 16-plexes (Fig. 4a; Supplementary Fig. 6). These samples were efficiently analyzed using GoDig with 2-h analyses targeting four different lists of proteins related to signaling or lipid biology. In total, each pathway or category list required ~60 h to measure the expression across the 480 livers. Remarkably, this is effectively 7.5 min per DO liver to measure protein expression across the entire pathway. An 18-plex reagent set is now available and would increase that throughput even more^8^.

We examined four pathways/categories of proteins in the DO mouse cohort (Figs. 4–7). We examined expression from proteins designated as belonging to a category termed “lipid metabolism.” Fifty proteins were selected based on the criterion that they had previously been identified as having a pQTL in the Chick et al. manuscript^35^. These pQTL were confirmed but at higher LOD scores using the GoDig assay (Fig. 4). We examined 50 proteins from the “kinase” category where no pQTL had been previously detected likely due to reduced power in the statistical test. We identified 13 pQTL from this experiment (Fig. 5).

More than 2200 lipid QTL have been identified using these same livers previously^19^. For most lipid QTL the protein driver was not identified. We applied GoDig in several cases to identify these drivers. For example, CES2H is an esterase that was identified through our pQTL mapping and mediation analysis as the potential driver for two unknown lipids from Linke and coworkers (Fig. 6)^19^. Based on the mass spectra for the unknown lipids and the known function of the *Ces2* gene family, we assigned the lipids as acylcarnitine species with molecular formulas C_14_H_26_NO_6_ (e.g., pimelylcarnitine) and C_13_H_24_NO_6_ (e.g., methylglutarylcarnitine) with their levels controlled by CES2H (Fig. 6; Supplementary Fig. 8). Finally, by using mediation analysis with the protein expression levels across the mice for the 220 targeted proteins, we identified that the gene *Gm4951* is found within a hotspot for pQTL from proteins annotated as being localized to lipid droplets (Fig. 7). Together with the separate experiment in AMl12 cell using different GM4951 perturbations (Fig. 7), these data point to GM4951 as a key regulatory protein for lipid droplet homeostasis.

In conclusion, GoDig combines real-time analytics and sample multiplexing to create a powerful targeted pathway analysis platform. We have provided here the GoDig technology platform and an accompanying targeted protein assay builder resource (https://wren.hms.harvard.edu/godig/) to greatly simplify assay generation. We have demonstrated its ability to simultaneously target entire pathways and categories of proteins in a single-shot experiment and to achieve sensitivities comparable to proteome-wide datasets. Our targeted investigation of protein categories across 480 DO mouse livers provided insights into genetic variation and its impact on lipid homeostasis. We envision that this technology can bridge the gap across genotype—proteotype—phenotype, placing proteotypic variation in its proper context that will empower both basic and applied biological research in the future.

## Methods

### Reagents

Reagents for tissue culture, including DMEM, fetal bovine serum (FBS), penicillin/streptomycin, and phosphate-buffered saline (PBS) were obtained from Gibco. Mass spectrometry-grade trypsin and Lys-C protease were purchased from ThermoFisher Scientific and Wako, respectively. Isobaric TMT reagents and the BCA protein concentration assay kit were from ThermoFisher Scientific. Empore-C18 material for in-house StageTips was acquired from 3 M and Sep-Pak cartridges were purchased from Waters. Sera-Mag Speed Beads (cat. nos. 45152105050350 and 65152105050350) were from GE Life Sciences (Marlborough, MA). All solvents used for liquid chromatography were purchased from VWR. Unless otherwise noted, all other chemicals were purchased from ThermoFisher Scientific.

### Mouse

All experiments involving mice were preapproved by an AAALAC-accredited Institutional Animal Care and Use Committee of the College of Agricultural Life Sciences (CALS) at the University of Wisconsin–Madison. The CALS Animal Care and Use Protocol number associated with the study is A005821, A.D. Attie, Principal Investigator. All mice were maintained in a temperature and humidity-controlled room on a 12 h light/dark cycle (lights on at 6:00 and off at 18:00), and provided water ad libitum.

### GoDig algorithm

GoDig was written in C# in the.NET Framework (v4.7.2). It has five modules, including data acquisition, real-time data visualization, data analysis, library construction and a Comet database builder. Spectral and elution libraries, target peptide lists, and the Comet database are custom-built and loaded to execute the GoDig method. A detailed workflow is illustrated in Supplementary Fig. 1 and a user guide is provided as Supplementary Note 1. GoDig utilizes the Fusion instrument API (freely available from ThermoFisher Scientific, https://github.com/thermofisherlsms/iapi). GoDig is freely available via a free user license for the Orbitrap Eclipse mass spectrometer platform (https://gygi.hms.harvard.edu/software.html).

### GoDig library construction

Data collected for library construction were searched using the open-source Comet search engine (ver. 2021.01.0)^44^. Depending on the sample type involved, UniProt human proteome database (downloaded December 21, 2018) or mouse ENSEMBLE proteome database (ver. 39 release 103) were used with contaminants and decoy sequences appended. Precursor error tolerance was 50 p.p.m. and fragment error tolerance was 0.02 Da. Static modifications include carboxyamidomethylation of Cys (+57.0215), as well as TMT11 (+229.1629) or TMTpro16 (+304.2071) on Lys side chains and peptide N-termini. A maximum of 3 methionine oxidation (+15.9949) events was allowed as variable modification. Search results were first filtered to a 1% peptide FDR using linear discriminant analysis employing a target-decoy strategy and further filtered to obtain a protein level FDR 1%^45–47^. Search results and raw files were exported and loaded in the library construction module of GoDig to build elution and spectral libraries. Only the best peptide spectral match (PSM) for each unique peptide (charge state considered) was included in the spectral library. Peptide PSM identification time was recorded in the elution library without additional retention time alignment between fractions. The elution library was later used to generate peptide elution bins based on user-specified bin width when setting up the targeted experiment.

### Real-time targeted proteomics assays using GoDig

GoDig was run on the data collection computer connected to the instrument. Experiments involving ADH1 peptides used 60-min (75-min method length) and 90-min (105-min method length) gradients, while experiments using the 4 human cell lines and DO mouse livers used a 105-min gradient (120-min method). Note that the instrument method only included Orbitrap MS1 scans (resolution of 120 K; mass range 400–1500 *m/z*; AGC target 2e5; max injection time of 50 ms; a single FAIMS CV of −60 V). All other scan types with CV values at −40, −60, and −80, including prescans, ion trap MS2, Orbitrap MS2, and Orbitrap MS3 scans were prompted for insertion via the API using the GoDig software. Peptide targets, elution and spectral libraries were loaded into GoDig.

GoDig listens to each collected MS1 spectrum in real time with the possibility of inserting five different scan types for different uses:

1. GoDig injects CV-specific prescans for each FAIMS CV value needed in addition to the one being collected by the instrument method. These are used to set the AGC.
2. Every 15 s, GoDig selects and fragments the top 6 precursors in the most immediate MS1 scan to collect fast ion trap MS2 scans. The resulting scans are searched and mapped to the elution library. Their elution bins are retrieved and used to calibrate the current elution point.
3. In parallel, GoDig sends ion trap PRM scans for targets within the calibrated elution window (±3 bin). Depending on the run length, bins represent between 15 and 30 s of elution time. The returned PRMs are matched in real time to theoretical fragments of specific targets (detection).
4. Upon matching a certain number of fragments in the ion trap PRM, GoDig prompts the collection of Orbitrap MS2 scans with a maximum injection time of 900 ms to accommodate weak signals. Fragment ions are matched and cosine-correlated to the library spectrum for the target peptide (identification).
5. When cosine similarity is >0.9 and more than 60% of the fragments with a relative abundance >50% in the library spectrum are present in the experimental spectrum, GoDig selects SPS-ions that pass a fixed purity threshold and inserts a custom SPS-MS3 scan (quantification). Maximum injection time is set to 2000 ms in order to accommodate even very weak signals.

The TMT reporter ion signals measured in each GoDig assay were extracted using the GoDig UI and exported to spreadsheets for downstream analysis in R (ver. 4.0.5) in RStudio^48,49^. Data formatting was performed using R/tidyverse and visualization was created using R/ggplot2^50,51^. Quantified peptides were filtered to require summed SN > 100 or >160 for TMT11- or TMTpro16-labeled samples, respectively. Likewise, SPS ion isolation purity was required to be >0.7 (70% of signals originated from the desired SPS ion in the window). MS3 quantification scans for the same peptide were summed. When more than three MS3 events were triggered for the same peptide, only the top three quantification events based on numbers of fragment ions matched were kept. Column normalization was performed to correct for different protein loading in each channel. For the experiments involving ADH1 peptides, known interference was measured in the blank channels without labeled ADH1 peptides and subtracted from the ADH1 measurements as baseline interference.

### DO animals and genotyping

DO mouse livers (*n* = 480) were obtained from the same mice used in previous studies^19,36,52^. Briefly, the DO mice were obtained at 4 weeks of age and maintained within the Department of Biochemistry animal vivarium at the University of Wisconsin. All DO mice were maintained on a HF/HS (high fat/ high sucrose) diet (44.6% kcal fat, 34% carbohydrate, and 17.3% protein) from Envigo Teklad (catalog number TD.08811). At ∼22 weeks of age, DO mice were euthanized with CO_2_, and their livers were harvested and flash frozen in LN2. Genotyping was performed on tail biopsies using the Mouse Universal Genotyping Array (GigaMUGA) [143,259 markers^53^] at Neogen (Lincoln, NE). Genotypes were converted to founder strain–haplotype reconstructions using R/DOQTL software^54^. We interpolated the GigaMUGA markers onto an evenly spaced grid with 0.02-cM spacing and added markers to fill in regions with sparse physical representation, resulting in 69,005 pseudomarkers. Further details regarding animal manipulation and genotyping can be found in the study by Keller et al.^36^.

### Founder strain animals

Livers from the 63 mice encompassing all 8 founder strains (A/J, C57BL/6J, CAST/EiJ, NOD/ShiLtJ, NZO/H1LtJ, PWK/PhJ, WSB/EiJ, 129S1/SvlmJ) were harvested from four females and four males of each strain (*n* = 8 mice per strain) fed the same diet as the DO mice above. Note there were only three mice of the CAST/EiJ male due to breeding difficulties. A mixture of the three male mouse livers was used to complete the 16-plex for this strain. In total, 64 samples were prepared, grouped, and labeled into four 16-plexes.

### GM4951 expression

Gateway-compatible pDONR murine Gm4951 (NM_001033767.3) was created by custom gene synthesis (GeneArt Express gene synthesis and cloning service, Invitrogen) and sequence verified via Sanger sequencing. The Gm4951 open reading frame was transferred to a gateway lentiviral destination vector with c-terminal FLAG-HA tag using LR clonase II according to the manufacturer’s instructions (Thermo Fisher Scientific), and lentiviral particles were prepared by transfecting HEK-293T cells with 2 µg of transfer vector and 0.5 µg of each helper plasmid encoding Gag-Pol, Rev, Tat, and VSV-G using polyethyleneimine (PEI). Cells were seeded into 60 mm plates one day prior to transfection. 48 h post transfection, viral supernatant was harvested, filtered, and frozen at −80  ^o^C. Thawed viral particles were used to infect mouse AML12 cells (ATCC). Forty-eight hours post-transduction, cells were selected with 1ug/mL puromycin (Sigma) to create stable expression lines.

### Preparation of cell line samples (related to Figs. 1, 2, 3)

Human RPE1 (#CRL-4000), U2OS (#HTB-96), HCT116 (#CCL-247), and HEK293T (#CRL-3216) cells were purchased from the American Type Culture Collection and grown in DMEM supplemented with 10% fetal bovine serum and 1% penicillin/streptomycin until 80% confluent. Cells were washed twice with ice cold PBS, pelleted and stored at −80 ^o^C until use. Cell pellets were processed as described previously^30^. In brief, cells were lysed by resuspension in lysis buffer followed by 10 passes through a 21-gauge syringe. Lysates were reduced with 5 mM tris(2-carboxyethyl)phosphine (15 min, room temperature [r.t.]) and alkylated with 10 mM iodoacetamide (30 min, r.t. in the dark). Excess iodoacetamide was quenched with 10 mM dithiothreitol (15 min, r.t.). Proteins were isolated by chloroform methanol precipitation, subsequently resuspended in 200 mM EPPS pH 8.5 (~1 mg/ml) and digested first with LysC for 12 h at r.t. shaking on a vortexer followed by a 6-h digestion at 37 ^o^C with trypsin. Protein digests were aliquoted to desired concentrations and labeled directly with separate TMT channels. The labeled peptides were then mixed and desalted on SepPak prior to basic pH fractionation or LC-MS analysis.

### Preparation of mouse tissue samples (related to Figs. 4–7)

10 μL of the homogenized tissue was mixed with 140 μL lysis buffer (8 M Urea, 100 mM EPPS, pH 8.5 with protease inhibitor) and lysed by 12 passes through a 21-gauge (1.25 inches long) needle. Protein concentrations were determined using the bicinchoninic acid (BCA) assay (ThermoFisher Scientific). Lysates were reduced with 5 mM tris(2-carboxyethyl)phosphine (15 min, r.t.) and alkylated with 10 mM iodoacetamide (30 min, r.t. in the dark). Excess iodoacetamide was quenched with 10 mM dithiothreitol (15 min, r.t. in the dark). Single-Pot, Solid-Phase-enhanced Sample processing (SP3) was used during protein isolation and digestion as described previously^55^. In brief, reactions were performed in 8-Strip PCR tubes using an 8-channel pipette. 10 µL (0.5 mg) of each Sera-Mag Speed Beads were added to 100 µg of protein in 100 µL total volume, as prepared above. Neat ethanol was added to a final concentration of 50%. The beads were carefully triturated 10 times. The samples were held to the magnet for 2 min and the supernatant was aspirated. The beads (with bound protein) were washed 3 times with 80% ethanol in the same manner. For protein digestion, we added 100 µL of 200 mM EPPS pH 8.5 and Lys-C overnight at room temperature, followed by trypsin for 6 h at 37 °C on an orbital shaker (Jitterbug Heated Microplate Shaker). Both enzymes were added a 1:100 protease-to-peptide ratio in the presence of beads. Protein digests were aliquoted to desired concentrations and labeled directly with separate TMTpro channels as described in Supplementary Data 2 for founder strains, and Supplementary Data 3 for DO mice.

### siRNA transfection in mouse AML12 hepatocyte

siRNAs targeting on GM4951 was purchased from Dharmacon (J-055897-11-0005). ON-TARGET plus Non-targeting Pool (Dharmacon) were used as control. AML12 cells were transfected in six-well dishes with 30 nM siRNA (final concentration) using Lipofectamine RNAiMAX Transfection Reagent (Thermo Fisher) according to the instructions of manufacturer. Cells were harvested after 60 h for proteome analysis experiment. Oleic acid was added to a final concentration of 200 µM, 32 h prior to the harvest.

### Basic pH reversed-phase fractionation

If fractionation was required, TMT-labeled mixtures (~200 µg) as 16-plexes were loaded onto an Agilent 300 Extend C18 column (3.5 μm particles, 2.1 mm ID and 250 mm in length). Peptides were separated using a 50 min linear gradient from 13 to 43% buffer B (90% acetonitrile, 10 mM ammonium bicarbonate, pH 8) at a flow rate of 0.25 ml/min. Fractions were collected into a 96-well plate and then were consolidated into 24 fractions. Samples were dried in a SpeedVac and each fraction was then desalted via StageTip^30^. The resulting desalted peptides were dried in a SpeedVacand and then resuspended in a solution containing 5% acetonitrile and 5% formic acid prior to LC-MS analysis.

### Analysis by liquid chromatograph coupled to mass spectrometry (LC-MS)

Unless otherwise noted, all mass spectrometry data were acquired using an Orbitrap Eclipse mass spectrometer in-line with a Proxeon NanoLC-1200 UPLC system and a FAIMS Pro device.

### LC-MS analysis for global protein expression profiling in mouse founder strains

Twenty-four basic pH HPLC fractions (see “Basic pH reversed-phase fractionation”) were collected for each of the four 16-plexes. Analysis of each fraction was performed with the real-time search (RTS) pipeline^14^. Samples were loaded on an in-house 100-µm capillary column packed with 30 cm of Accucore 150 resin (2.6 μm, 150 Å; Thermo Fisher Scientific). Twelve out of the 24 basic pH fractions per TMTpro16 set were separated using a 120-min method, while the remaining 12 were separated using a 90-min method. Data were collected using an RTS-powered SPS-MS3 method with the mass spectrometer alternating between three compensatory FAIMS voltages (−80, −60 and −40 V). MS1 scans were collected in the Orbitrap with a resolution setting of 120 K, a 50% AGC target and a maximum injection time of 50 ms. MS2 scans were acquired in Top Speed mode with a cycle time of 1.2 s. MS2 AGC was set at 100%, and a maximum injection time of 35 ms was allowed. Dynamic exclusion was enabled with an exclusion duration of 120 s and a mass tolerance of ±7 p.p.m. and only one charge state was allowed to trigger MS2 scans per precursor. Resulting MS2 scans were searched in real time using the Comet search engine (https://uwpr.github.io/Comet/)^14,44^ and the returned peptides were filtered using simple initial filters that included: not matching a reversed-sequence, maximum PPM error <10, minimum cross-correlation of 0.5, minimum deltaCorr of 0.10 and minimum peptide length of 7. If peptide spectra matched those criteria above, an SPS–MS3 scan was inserted using up to 10 *b-* and *y-*type fragment ions as precursors with an AGC of 200 K for a maximum of 250 ms, with a normalized collision energy setting of 55.

### LC-MS analysis to construct libraries for TMT11-labeled ADH1 experiments

Libraries for benchmarking experiments using TMT11-labeled ADH1 peptides in the HCT background were built using a 90-min gradient (105 min method length). Data were collected using a high-resolution MS2 method with the mass spectrometer alternating between three compensatory FAIMS voltages (−80, −60 and −40 V). MS1 scans were collected in the Orbitrap with a resolution setting of 120 K, a 50% AGC target and a maximum injection time of 50 ms. MS2 scans were acquired in the Orbitrap (15 K resolution) in Top Speed mode with a cycle time of 1 s, a collision energy of 35, and an isolation width of 0.5. MS2 AGC was set at 100%, and a maximum injection time of 100 ms was allowed. Dynamic exclusion was enabled with an exclusion duration of 90 s and a mass tolerance of ±7 p.p.m.

### LC-MS analysis to construct libraries for GoDig analysis of 4 TMTpro16-labeled human cell lines

24 basic pH HPLC fractions (see “Basic pH reversed-phase fractionation”) were loaded and separated on an in-house 100-µm capillary column packed with 30 cm of Accucore 150 resin (2.6 μm, 150 Å; Thermo Fisher Scientific). LC separation used a 105-min gradient (120 min method length). Data were collected using a high-resolution MS2 method with the mass spectrometer alternating between three compensatory FAIMS voltages (−80, −60, and −40 V). MS1 scans were collected in the Orbitrap with a resolution setting of 120 K, a 50% AGC target, and a maximum injection time of 50 ms. MS2 scans were acquired in the Orbitrap (15 K resolution) Top Speed mode with a cycle time of 1 s, a collision energy of 34, and an isolation width of 0.5. MS2 AGC was set at 100%, and a maximum injection time of 150 ms was allowed. Dynamic exclusion was enabled with an exclusion duration of 120 s and a mass tolerance of ±7 p.p.m., and only one charge state was allowed to trigger MS2 scans per precursor.

### LC-MS analysis to construct libraries for TMTpro16-labeled DO mouse samples

24 basic pH HPLC fractions were obtained by pooling a quarter of each of the corresponding fractions collected for the four founder-strain 16-plexes. Samples were loaded and separated on an in-house 100-µm capillary column packed with 30 cm of Accucore 150 resin (2.6 μm, 150 Å; Thermo Fisher Scientific). LC separation used a 105-min gradient (120 min method length). Data were collected using a high-resolution MS2 method with the mass spectrometer alternating between three compensatory FAIMS voltages (−80, −60 and −40 V). MS1 scans were collected in the Orbitrap with a resolution setting of 120 K, a 50% AGC target and a maximum injection time of 50 ms. MS2 scans were acquired in the Orbitrap (15 K resolution) in Top Speed mode with a cycle time of 1 s, a collision energy of 34, and an isolation width of 0.5. MS2 AGC was set at 100%, and a maximum injection time of 150 ms was allowed. Dynamic exclusion was enabled with an exclusion duration of 120 s and a mass tolerance of ±7 p.p.m., and only one charge state was allowed to trigger MS2 scans per precursor.

### Data analysis for global protein expression profiling in mouse founder strains

Data were searched using the open-source Comet search engine (ver. 2019.01.5)^44^ with the mouse Ensembl proteome database (ver. 39 release 103) with contaminants and reverse decoy sequences appended. Precursor error tolerance was 50 p.p.m. and fragment error tolerance was 0.9 Da. Static modifications include Cys carboxyamidomethylation (+57.0215) and TMTpro16 (+304.2071) on Lys side chains and peptide N-termini. A maximum of 3 methionine oxidation (+15.9949) events was allowed as variable modification. Search results were first filtered to a 1% peptide FDR using linear discriminant analysis employing a target-decoy strategy and further filtered to obtain a protein level FDR 1%^45–47^. TMT reporter ion signal was extracted by allowing a 0.003 Da mass tolerance and signal-to-noise (SN) ratios were calculated for each channel. A limit of quantification filter was implemented by requiring a minimum summed SN > 100 for TMT11-labeled samples and >160 for TMTpro16-labeled samples. Column normalization was performed to correct for different protein loading in each channel. Results were further analyzed in R (ver. 4.0.5) in RStudio^48,49^. Data formatting was performed using R/tidyverse and visualization was created using R/ggplot2^50,51^ and RColorBrewer. Protein abundance was modeled with linear regression with strain and sex as covariates. Only proteins quantified in at least three biological replicates were included. Benjamini-Hochberg adjusted *p* values were obtained and proteins with an adjusted *p* < 0.05 were considered to be differentially regulated.

### Protein QTL mapping

Protein abundance values were first normalized as ratios to the group (plex) mean and then log2 transformed. QTL were mapped using the *scan1* function in qtl2 R package^56^, which fits a linear mixed effect model for each protein as an outcome variable at putative QTL individually across the genome. Sex and batch (breeding generation of the mice) were included as additive covariates and a random polygenic term to account for genetic relatedness was included in the mapping^35^. The QTL term that is tested is modeled in terms of scaled probabilistic counts (i.e., dosages) of the 8 founder haplotypes at the genomic position. The founder allele effects at each QTL were generated using the *scan1coef* function, which extracts the 8 founder haplotype regression coefficients.

### Statistical analysis of pQTL candidates

Significance thresholds for QTL mapping were established by performing 10,000 permutations using the function *scan1perm* in the qtl2 R package^56^. Permutation-derived empirical *p*-values were calculated as the proportion of permutation LOD scores that were greater than the detected QTL LOD score, and then converted to *q*-values with the *q* value R package^57^. The significance threshold for declaring a QTL was set at *q* value < 0.1.

### Mediation analysis

Mediation was performed using the intermediate R package^35^. Individual candidate mediator proteins were included as an additive covariate in the QTL mapping model (with polygenic term excluded) fit at the peak locus and the regression was performed again. The assumption was that by including the covariate, true mediators should cause a substantial decrease in the QTL LOD score. The proportion reduction in QTL LOD score is recorded as a summary. When many candidate mediators were evaluated for the same pQTL, Z-scores were calculated across the LOD score reductions to capture information on the distribution of mediation scores.

### Reporting summary

Further information on research design is available in the Nature Portfolio Reporting Summary linked to this article.

## Supplementary information

Supplementary Information

Description of Additional Supplementary Files

Dataset 1

Dataset 2

Dataset 3

Dataset 4

Dataset 5

Dataset 6

Dataset 7

Reporting Summary

## Data Availability

This project has generated many types of data and code that are available for distribution via numerous venues. Items not listed here will be provided by the Lead Contact upon reasonable request. The mass spectrometry data profiling liver proteomes from 8 mouse founder strains have been deposited at the ProteomeXchange Consortium with the dataset identifier PXD029461. Global proteome data in founder mouse strains generated during this study are also available using the viewer on the Gygi lab website (https://gygi.hms.harvard.edu/resources.html). The mass spectrometry data profiling 4 human cell lines have been deposited at the ProteomeXchange Consortium with the dataset identifier PXD033643. The mass spectrometry data profiling AML12^*WT*^ and AML12^*GM4951*^ with and without siGM4951 have been deposited at the ProteomeXchange Consortium with the dataset identifier PXD033679. The 297 raw files from GoDig experiments have been deposited at the MassIVE repository (https://massive.ucsd.edu/ProteoSAFe/static/massive.jsp) with the dataset identifier MSV000090110 [10.25345/C57H1DR40]. The liver lipidomics dataset used here was published by Linke et al. ^19^ and has been deposited in Chorus (https://chorusproject.org/anonymous/download/experiment/a639bcc5602c441c9a1df94f4340d626). In addition, source data for individual plots can be found in Source Data. Source data are provided with this paper.

## Source data

Source Data

## Code availability

GoDig relies on real-time access to spectral data which is provided under the free license provided by the Thermo Scientific Fusion API (https://github.com/thermofisherlsms/iapi). GoDig uses the API to control Orbitrap Fusion-based instruments. To enable GoDig, the RTS Comet functionality (ver. 2021.01.0) is required and has already been released (https://uwpr.github.io/Comet/). The GoDig software is freely available via a Recipient Agreement for the Orbitrap Eclipse or the Orbitrap Ascend mass spectrometer platforms with an API license. The Recipient Agreement and the instruction to obtain the API license can both be obtained at https://gygi.hms.harvard.edu/software.html. GoDig is enabled through the API framework such that the source code is not available. Targeted assays can be built using the GoDig targeted protein assay builder (https://wren.hms.harvard.edu/godig/).
