# Supplementary material for: Sample multiplexing-based targeted pathway proteomics with real-time analytics reveals the impact of genetic variation on protein expression
Source: Nat Commun. 2023 Feb 2;14:555. doi: 10.1038/s41467-023-36269-7 (PMC9894840; doi:10.1038/s41467-023-36269-7)
Supplement: Supplementary file 1 — Supplementary Information [file 41467_2023_36269_MOESM1_ESM.pdf]

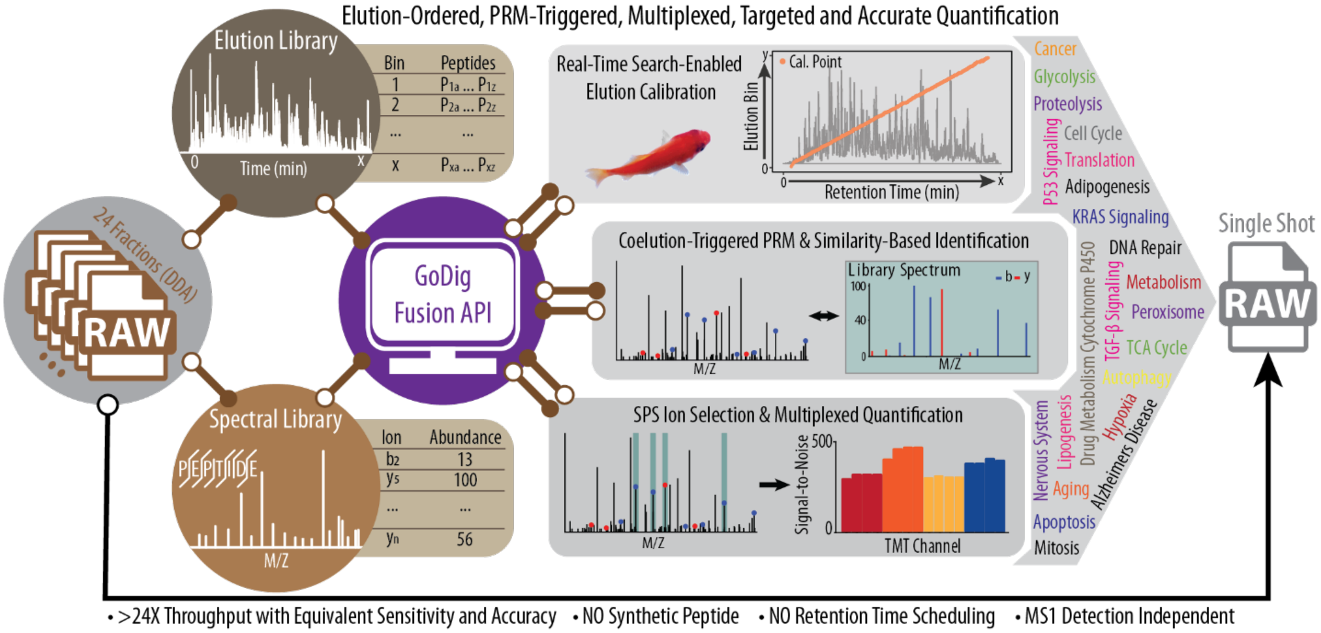

Supplementary Figure 1. Detailed GoDig design and workflow. GoDig extracts relative elution information and peptide spectral matches to construct elution and spectral libraries. The libraries along with a list of target peptides are loaded into GoDig for method execution. GoDig listens to the MS1 scans collected by a mass spectrometer and directly inserts various scan types: 1) optional MS1 prescans for AGC calculation if the FAIMS Pro device is used, 2) periodic (e.g., every 15 s) MS2 scans fragmenting the top peptide precursors in an MS1 scan which are searched in real-time to calibrate the elution position, 3) continuous insertion of fast ion trap PRM (IT-PRM) scans to monitor peptide targets whose elution bins are within the current predicted elution window (calibrated bin ± a predefined bin range), 4) high-resolution Orbitrap MS2 (OT-MS2) scans triggered by matched IT-PRM to ensure peptide identification, 5) custom SPS-MS3 scans with ultra-long injection times for quantification.

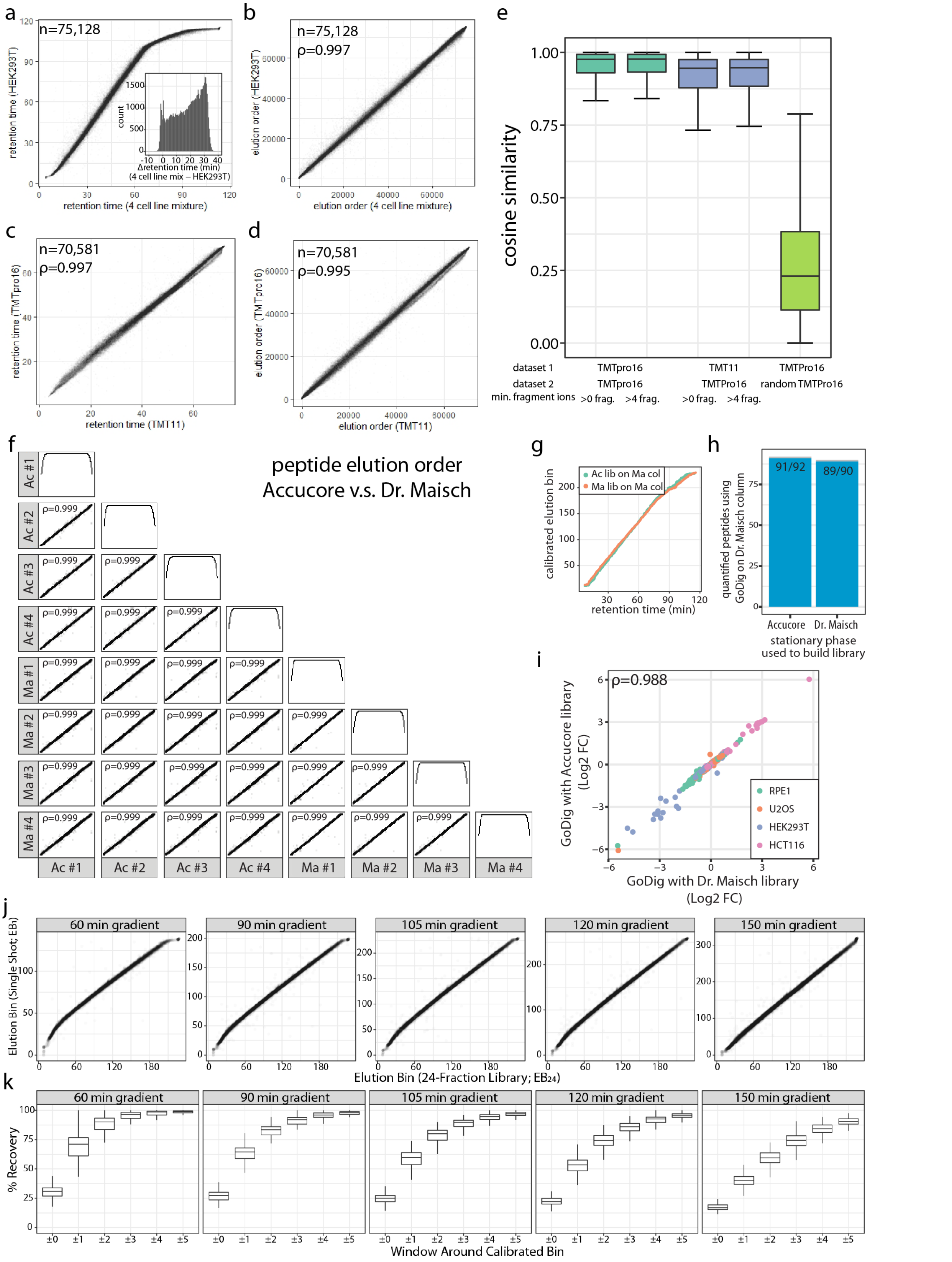

Supplementary Figure 2. GoDig relies on the robust relative elution profile and spectral library to perform targeted experiments.

a-b Relative elution profile is robust to different sample types and TMT reagents. **a** Retention time scatterplot of common peptides identified in a HEK293T peptide sample (different gradient and MS system) compared to the 4 human cell line

mixture used in Figure 2. The inset shows the ΔRT. Since different LC systems, columns and gradients were used, retention times varied by up to 40 min, making retention time scheduling infeasible. **b** Despite RT shift in **a** the relative elution order stayed highly consistent with a spearman ρ=0.997. **c-d** Retention time and elution order align well between TMTpro16- and TMT11-labeled peptides. The two labeling reagents have a similar impact on peptide retention. **e** Cosine similarity for peptide spectra across different datasets. Peptides from two TMTpro16-labeled datasets present high consistency with a median cosine distance=0.98 (n=105,281 peptide pairs). Even peptides labeled with either TMTpro16 or TMT11 reagents still have a highly consistent pattern (median cosine distance=0.95; n= 131,673 peptide pairs). Removing spectra with <5 fragments slightly improved cosine similarity. Randomly comparing spectra from TMTpro16-labeled peptides only has a median cosine similarity 0.23 (n= 203,537 peptide pairs). Bottom border, interior line, and top border in the box plot represent the 1st quartile, median, and 3rd quartile, respectively. Source data are provided in the Source Data file. **f-i** Peptide elution and spectral libraries are transferable across two commonly used C18 stationary phases, Accucore (solid core, 2.6 µm) and Dr. Maisch (ReproSil-Pur 120 C18-AQ, 2.4 µm; cat no: r124.aq.0001). **f** TMTpro16-labeled 4 cell line sample was analyzed using a 120 min gradient on a 30 cm column packed with either Accucore (Ac) or Dr. Maisch (Ma) particles. Four replicates were acquired on either stationary phase with a shotgun method. The elution order of identified peptides stayed consistent regardless of the packing material. **g** Two peptide libraries were built with either the data acquired on Accucore or Dr. Maisch and used with GoDig to perform targeted quantification of ~90 peptides. The real-time elution profiles are in agreement despite different libraries used, suggesting libraries built with the two commonly used stationary phases are interchangeable. **h** Number of successfully quantified peptides using either library in the GoDig experiment on the Dr. Maisch column. 99% of peptides were quantified in a single shot. **i** Peptide quantification was consistent whether the Accucore library or Dr. Maisch library was used on the Dr. Maisch column. **j-k** Peptide elution information gathered from deeply fractionated dataset can be used to calibrate the elution position in single shot analysis of unfractionated experiment. An elution library was built using the data from 24 fractions of the 4 human cell line sample with a 2-hr method (105-min gradient). On a different day and with a different LC column, 5 single-shot experiments using different gradients were performed with the unfractionated sample. Elution bin position was calculated for each peptide identified in the experiments. **j** Elution bin alignment between the single-shot experiments (EB_1) and the 24-fraction library (EB_{24}). Although the gradients and exact retention times were different, the relative elution order remained consistent. **k** Recovery of peptides detected in the single-shot analysis based on the bin calculated for elution. Including a window of a few bins on either side of the predicted elution bin resulted in >90% recovery of peptides within the window, suggesting that peptides elute very near their predicted position. Bottom border, interior line, and top border in the box plot represent the 1st quartile, median, and 3rd quartile, respectively. We note that this in-silico bin matching is purely based on identification time. In reality, the chromatographic peak width is wider, and the actual recovery is likely even better.

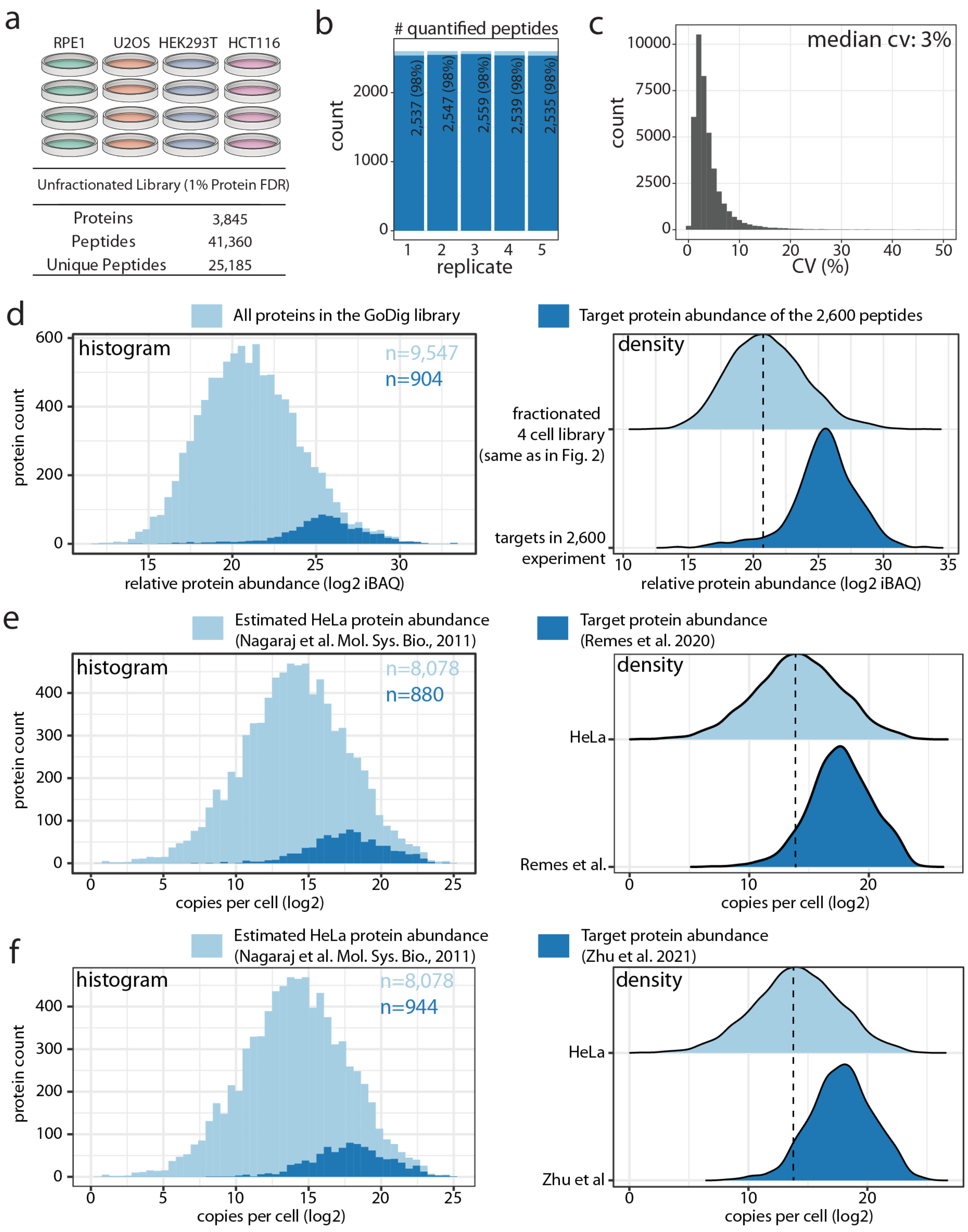

Supplementary Figure 3. Example of targeting 2,600 abundant peptides in a single. GoDig targeted analysis of peptides across 4 human cell lines (16 samples) with a small library constructed from unfractionated sample, related to Figure 2, 3. a Biological quadruplicates of RPE1, U2OS, HEK293T and HCT116 were processed and labeled with TMTpro16. To construct the libraries for elution prediction and spectral matching, the unfractionated sample was analyzed in duplicate with a 2-hr gradient. With no fractionation, only 3,845 proteins were included in this library. **b** The library was used to target 2,600 peptides with GoDig. Five technical replicates were collected and >98% of peptides were successfully quantified in each replicate. **c** Coefficient of variation (CV) of the quantitative values among technical replicates for each peptide. The median CV was 3%. **d** Protein abundance estimated using iBAQ values of the targeted proteins. 2,600 targeted peptides are mapped to 904 proteins and the iBAQ values for targets and background proteins are calculated with the fractionated 4 cell line samples as in Fig. 2. **e** The target protein abundance from Remes et al.[1] is mapped to corresponding HeLa background reported by Nagaraj et al.[2] 1,489 target peptides from Remes et al are

assigned to 880 proteins using the unique-parsimony rule. **f** The target protein abundance from Zhu et al [3] is mapped to corresponding HeLa background reported by Nagaraj et al.[2] 1,857 target peptides from Zhu et al are assigned to 880 proteins using the unique-parsimony rule.

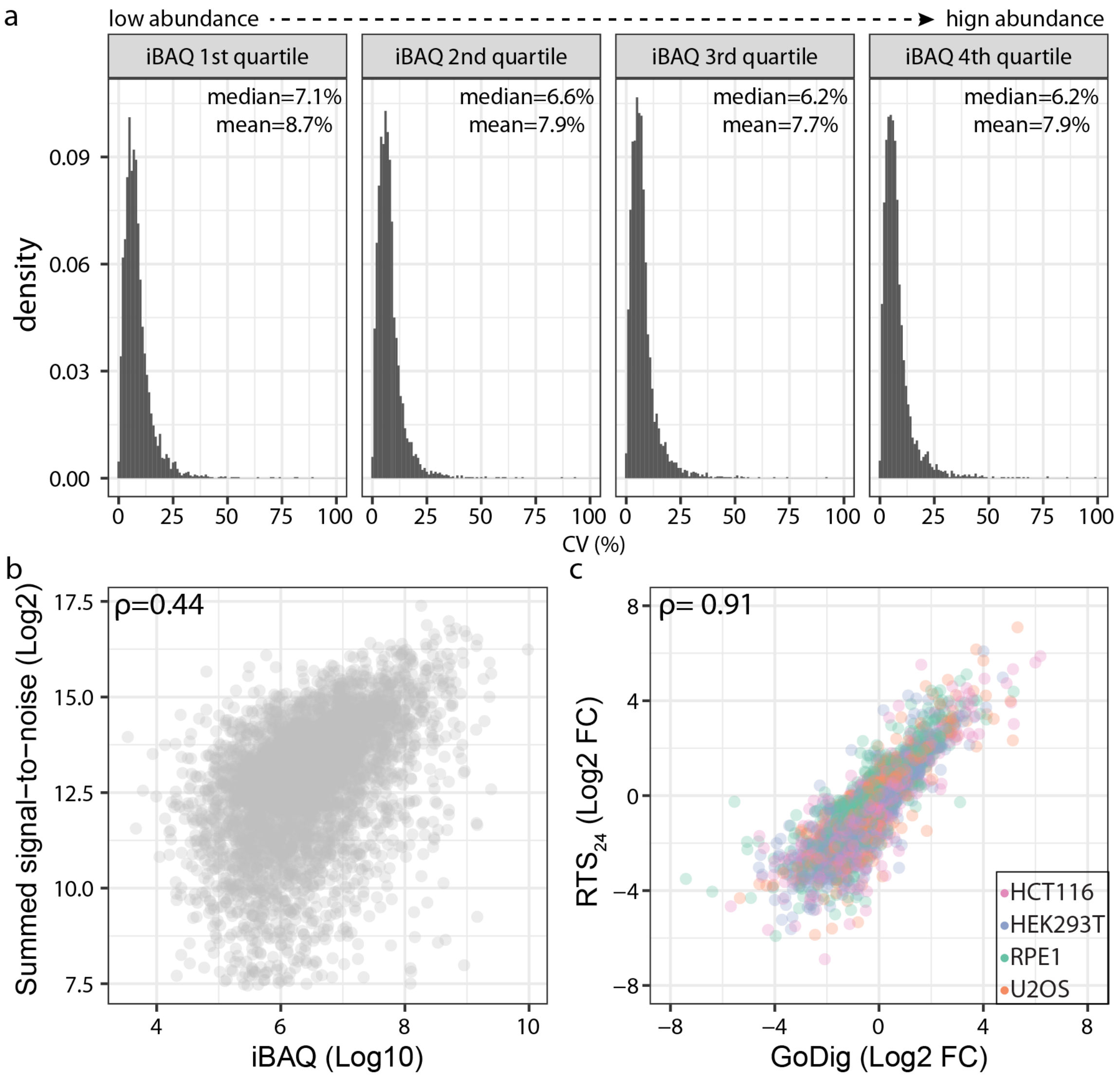

Supplementary Figure 4. Quantification of 4,000 randomly selected proteins (20 sets x 200 proteins), related to Figure 3e, f. a Protein targets are binned to quartiles based on their iBAQ[4,5] values, and CV values among biological replicates are calculated. Despite a subtle decrease in CV with increasing protein abundance, all median and mean CV values are <10%. **b** Summed signal noise is moderately correlated with protein abundance (iBAQ), with a spearman ρ=0.44. Source data are provided in the Source Data file. **c** Quantification generate by GoDig is in good agreement with deep fractionated dataset with a spearman ρ=0.91. The fold change represents the ratio between the mean of a single cell line and the mean of the other 3 cell lines. Source data are provided in the Source Data file.

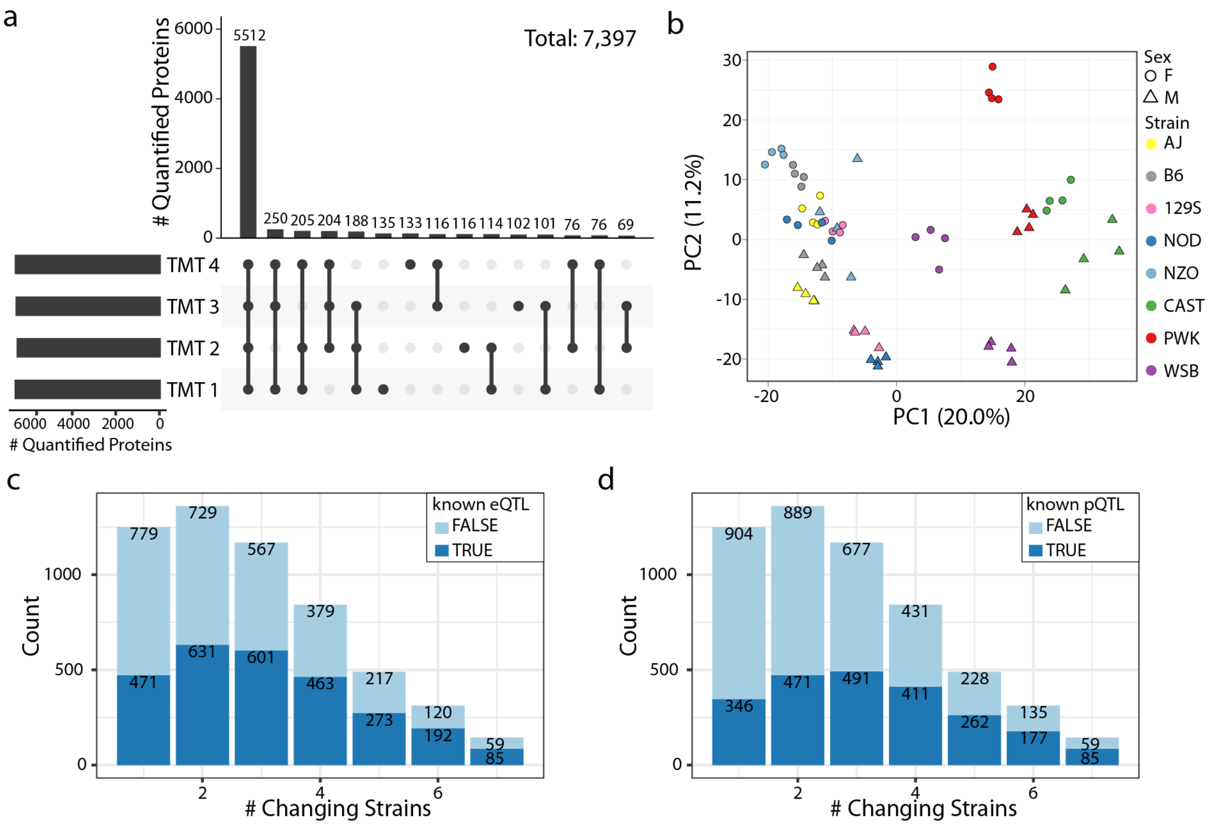

Supplementary Figure 5. Proteome-wide profiling of livers from 8 founder strains (n=8 for each strain; 4 male, 4 female), related to Figures 4, 5, 6, 7. a Number of quantified proteins in each TMT set. 7,397 proteins were quantified in total, and 5,512 were quantified across all samples. **b** Principal component analysis (PCA) reveals large scale differences based on strain and sex. The wild-derived strains (CAST, PWK and WSB) notably separate from the more traditional laboratory strains. **c** Number of proteins with significant changes are binned by the number of changing strains, relative to the B6 mouse, and colored by whether the protein has an eQTL annotation in Chick et al.[6] **d** Number of proteins with significant changes are binned by the number of changing strains, relative to B6 mouse, and colored by whether the protein has a pQTL annotation in Chick et al.[6] Protein abundance was modeled with linear regression with strain and sex as covariates. Benjamini-Hochberg adjusted p-values were obtained and proteins with an adjusted $p<0.05$ were considered to be differentially regulated.

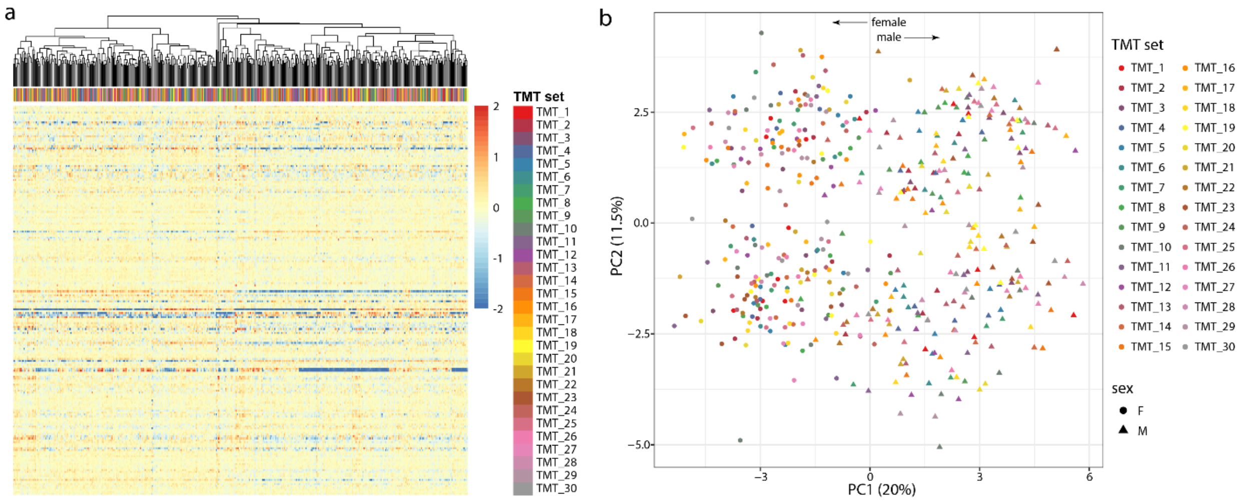

Supplementary Figure 6. Hierarchical clustering (a) and principal component analysis (b) of the 220 proteins quantified across 480 DO mouse livers. Samples were clustered regardless of their TMT plex origin, and primarily separated on the PCA plot based on the sex of the animal (PC1).

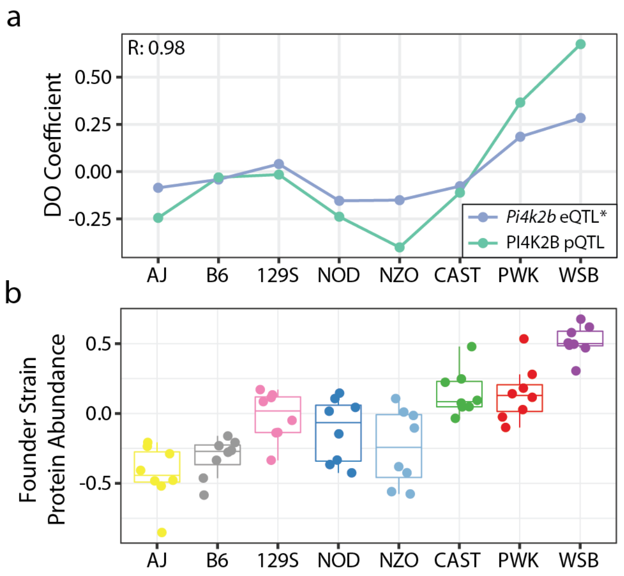

Supplementary Figure 7. PI4K2B is an example of the transcript-to-protein regulation model, related to Figure 4. a The inferred allele effects for the *Pi4k2b* eQTL and the PI4K2B pQTL are similar. Source data are provided in the Source Data file. **b** Founder strain PI4K2B liver protein abundance (n=8). Bottom border, interior line, and top border in the box plot represent the 1st quartile, median, and 3rd quartile, respectively. *eQTL data from Chick et al.[6] Source data are provided in the Source Data file.

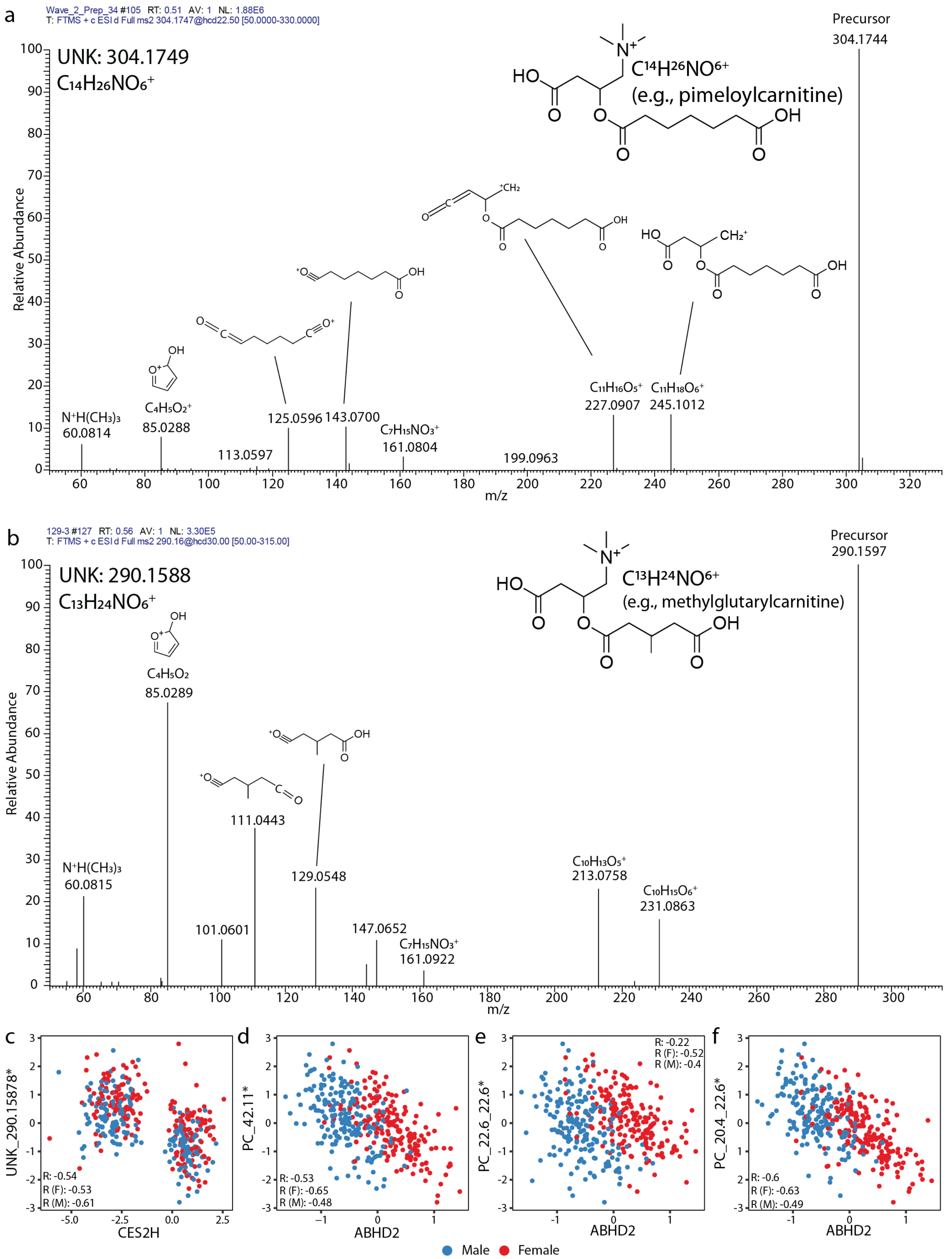

Supplementary Figure 8. CES2H and ABHD2 regulate abundance of several lipid species and the regulator relationship assist annotation of unknown lipid species, related to Figure 6.

a-b Annotated spectra for UNK:304.17487 and UNK:290.15878. Diagnostic ions for acylcarnitines were identified the spectra were identified as acylcarnitine species with molecular formulas $C_{14}H_{26}NO_6^+$ (e.g., pimeloylcarnitine) and

$C_{13}H_{24}NO_6^+$ (e.g., methylglutarylcarnitine). Spectra extracted from Linke et al[7]. **c-f** Examples of co-regulated lipid and protein abundance across DO mouse livers. Points are colored based on sex. Overall and sex-specific Pearson correlations were annotated in each panel. Protein abundances on the x-axis represent the log2 fold change from the group mean. *Lipid abundance from Linke et al[7].

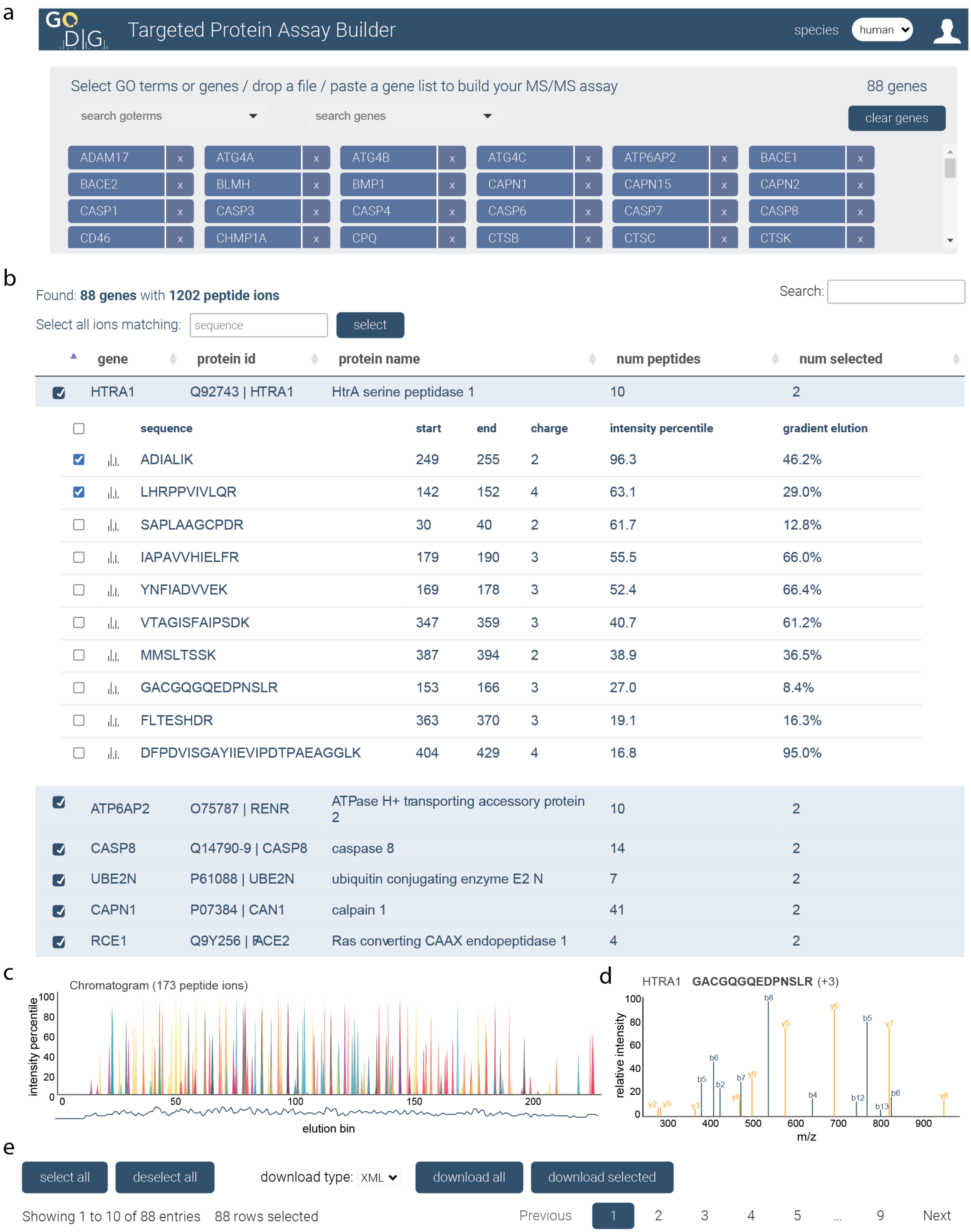

Supplementary Figure 9. GoDig targeted protein assay builder website. a The GoDig assay builder website allows the building of assays for individual genes, gene lists and GO categories. **b** All possible surrogate library peptides are shown for the targeted genes with information needed for selection. In this case, the two peptides with the highest precursor intensity percentile are automatically selected. **c** The in-silico predicted chromatogram of the selected peptides. Intensity percentile is calculated from the library's measured precursor intensity. The blue line below the x-axis shows the number of peptides that would be targeted for PRM analysis at any moment of the assay for the 88 selected proteins. **d** The MS2 library spectrum (used for cosine similarity matching) for each peptide can be displayed by clicking the spectrum symbol in front of each peptide entry.

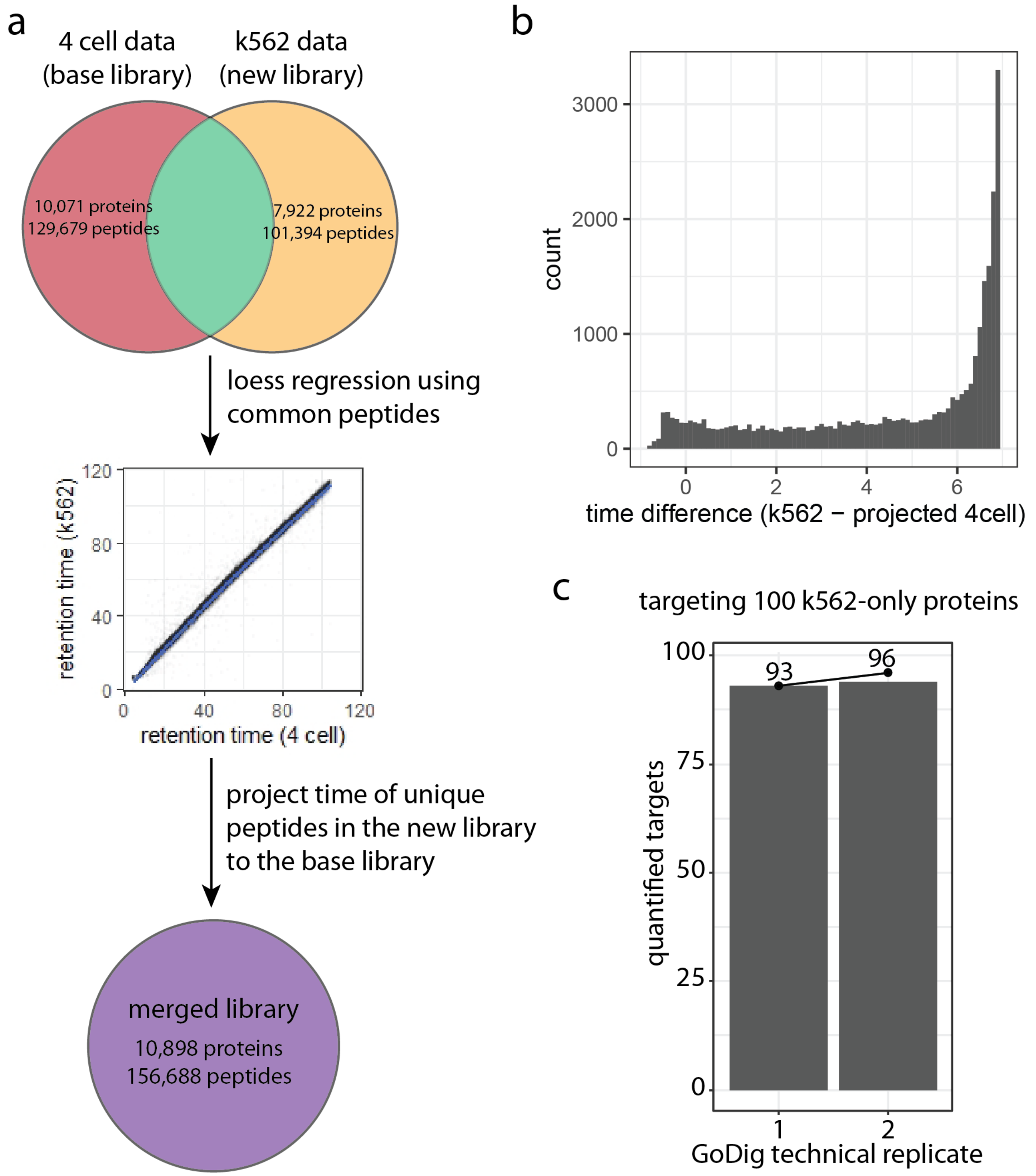

Supplementary Figure 10. Expanding the peptide/proteome library. The original library built with TMTpro16-labeled 4 human cell lines can be further expanded to include data from different samples. **a** To expand the 4 cell library (base library), a dataset collected using peptides derived from human K562 cells (new library) was included. Peptides shared between them were aligned and used to perform the loess regression. The regression was then used to project retention times of those peptides specific to the new library into the base library. All peptides then were used to build a merged library including 10,898 proteins and 156,688 unique peptides. **b** Retention time differences between the original times in the new library and the projected times in the merged library for peptides only detected in the K562 dataset. **c** 100 proteins (300 peptides) unique to the K562 dataset were selected and targeted using GoDig and the merged library on a TMTpro16-labeled K562 sample. Two technical replicates were collected and GoDig quantified 93 and 94 proteins, respectively. Cumulatively GoDig quantified 96 proteins.

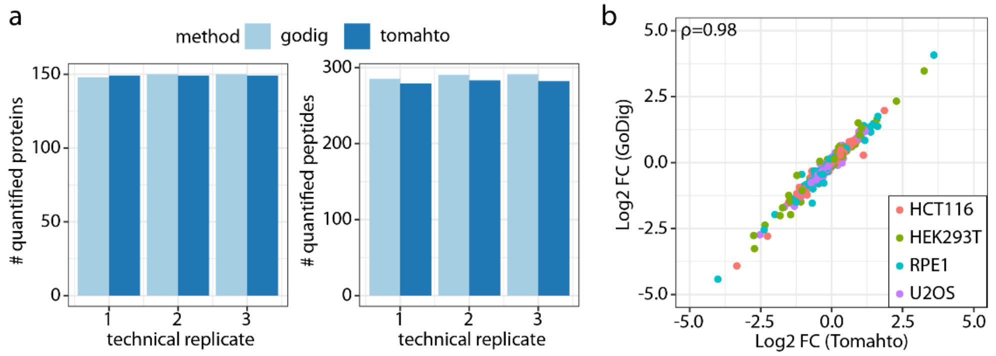

Supplementary Figure 11. Quantitative comparison between GoDig and Tomahto. To compare the quantitative accuracy between GoDig and Tomahto[8], we selected 150 proteins (300 peptides; 2 peptides per protein) that have the same peptides in our GoDig library and the JPT SpikeMix™ ABRF synthetic peptide standard (JPT peptide, catalogue # SPT-ABRF-POOL-L). For Tomahto, 100 fmol of the synthetic peptides were loaded on column along with 1 µg the TMTpro16-labeled 4-cell-line sample, whereas for GoDig no synthetic peptides were used. Each sample was analyzed using Tomahto or GoDig in technical triplicate. **a** Number of quantified proteins and peptides. **b** Correlation between GoDig and Tomahto protein quantification. The fold change represents the ratio between the mean of a single cell line and the mean of the other 3 cell lines. The Spearman correlation is 0.98.

Supplementary References

1. Remes, P. M., Yip, P. & MacCoss, M. J. Highly Multiplex Targeted Proteomics Enabled by Real-Time Chromatographic Alignment. *Anal. Chem.* **92**, 11809–11817 (2020).
2. Nagaraj, N. *et al.* Deep proteome and transcriptome mapping of a human cancer cell line. *Molecular Systems Biology* **7**, 548 (2011).
3. Zhu, H. *et al.* PRM-LIVE with Trapped Ion Mobility Spectrometry and Its Application in Selectivity Profiling of Kinase Inhibitors. *Anal. Chem.* **93**, 13791–13799 (2021).
4. Schwanhäusser, B. *et al.* Global quantification of mammalian gene expression control. *Nature* **473**, 337–342 (2011).
5. Schaab, C., Geiger, T., Stoehr, G., Cox, J. & Mann, M. Analysis of High Accuracy, Quantitative Proteomics Data in the MaxQB Database *. *Molecular & Cellular Proteomics* **11**, (2012).
6. Chick, J. M. *et al.* Defining the consequences of genetic variation on a proteome-wide scale. *Nature* **534**, 500–505 (2016).
7. Linke, V. *et al.* A large-scale genome–lipid association map guides lipid identification. *Nat Metab* **2**, 1149–1162 (2020).
8. Yu, Q. *et al.* Sample multiplexing for targeted pathway proteomics in aging mice. *Proceedings of the National Academy of Sciences* **117**, 9723–9732 (2020).

Supplementary Note 1

Quick guide to GoDig

Qing Yu,[1] Xinyue Liu,[1] Mark P Keller,[2] Jose Navarrete-Perea,[1] Tian Zhang,[1] Sipei Fu,[1] Laura P Vaites,[1] Steven R Shuken,[1] Ernst Schmid,[3] Gregory R Keele,[4] Jiaming Li,[1] Edward L Huttlin,[1] Edrees H Rashan,[2] Judith Simcox,[2] Gary A Churchill,[4] Devin K Schweppe,[5] Alan D Attie,[2] Joao A Paulo,[1] Steven P Gygi[1,*]

[1]Department of Cell Biology, Harvard Medical School, Boston, MA 02115, USA.
[2]Department of Biochemistry, University of Wisconsin-Madison, Madison, WI 53706, USA
[3]Department of Biological Chemistry and Molecular Pharmacology, Harvard Medical School, Boston, MA 02115, USA.
[4]The Jackson Laboratory, Bar Harbor, Maine 04609, USA
[5]Department of Genome Sciences, University of Washington, Seattle, WA 98105, USA

Download Tomahto

- Prerequisite: Thermo API license (https://github.com/thermofisherlsms/iapi)
- Download website: https://gygi.med.harvard.edu/software (also smarttmt.org).
- Orbitrap Tune software: 3.5 or later version

Smart-TMT

Real-Time-Search

The Real-Time Search** (RTS-MS^3) provides real-time (<5 ms / spectrum) spectral identification and triggers SPS-MS^3 scans that utilize assigned and pure fragment ions for accurate quantitation.Time consuming SPS-MS^3 spectra are only acquired after confident peptide identification, greatly increasing the number of peptides interrogated and reducing the effects of isobaric interference. **Try it!**

Tomahto

The Tomahto software provides real-time instrument control and decision making. Tomahto enables simplied implementation of TOMAHAQ targeted assay. It provides an array of functionalities including MS1 peak detection, MS2 real-time peak matching (RTPM), MS2 fragmentation pattern match, SPS ion purity filter, MS3 automatic gain control (AGC), MS3 quant scan insertion, and target peptide close-out. In addition to controlling data acquisition, it also allows real-time data visualization and post-acquisition analysis. **Try it!**

GoDig

The GoDig software is a real-time analytics platform that enables next-generation TMT-based multiplexed targeted proteomics. It obviates the need for making internal peptide standards and tedious method curation. As a result, it essentially makes targeting nearly any prviously detected peptides possible. One only needs a list of peptides and GoDig will perform real-time elution calibration and spectral matching to identify the targets, and prompt SPS-MS3 scans to quantify them down to attomole level. **Try it!**

General considerations for trigger peptide selection

1. Should be unique to the protein of interest
2. Should avoid missed cleavages to minimize artifact caused by sample preparation
3. Should not contain methionine to avoid artifact caused by oxidation
4. If a peptide contains cysteines, an extra step needs to be taken to reduce/alkylate the free cysteines using the same protocol as the endogenous protein sample preparation
5. Preferred peptide length is 8~20 amino acids to ensure proper hydrophobicity
6. We prefer to have 2 peptides per target protein as a fail-safe measure

How to acquire data using GoDig.exe

1. Connect instrument
2. Select target peptide list (.csv)

Example of input .csv file

GeneSymbol	Peptide
Abhd1	YTSVAFGYK
Abhd1	VLDVDFAIK
Abhd10	SELPNLAYK
Abhd10	IPYSFIK
Abhd11	LNLDTLAQHLDK
Abhd11	TNFNSLAK
Abhd11	VLTVDAR
Abhd12	VPYFIDLK

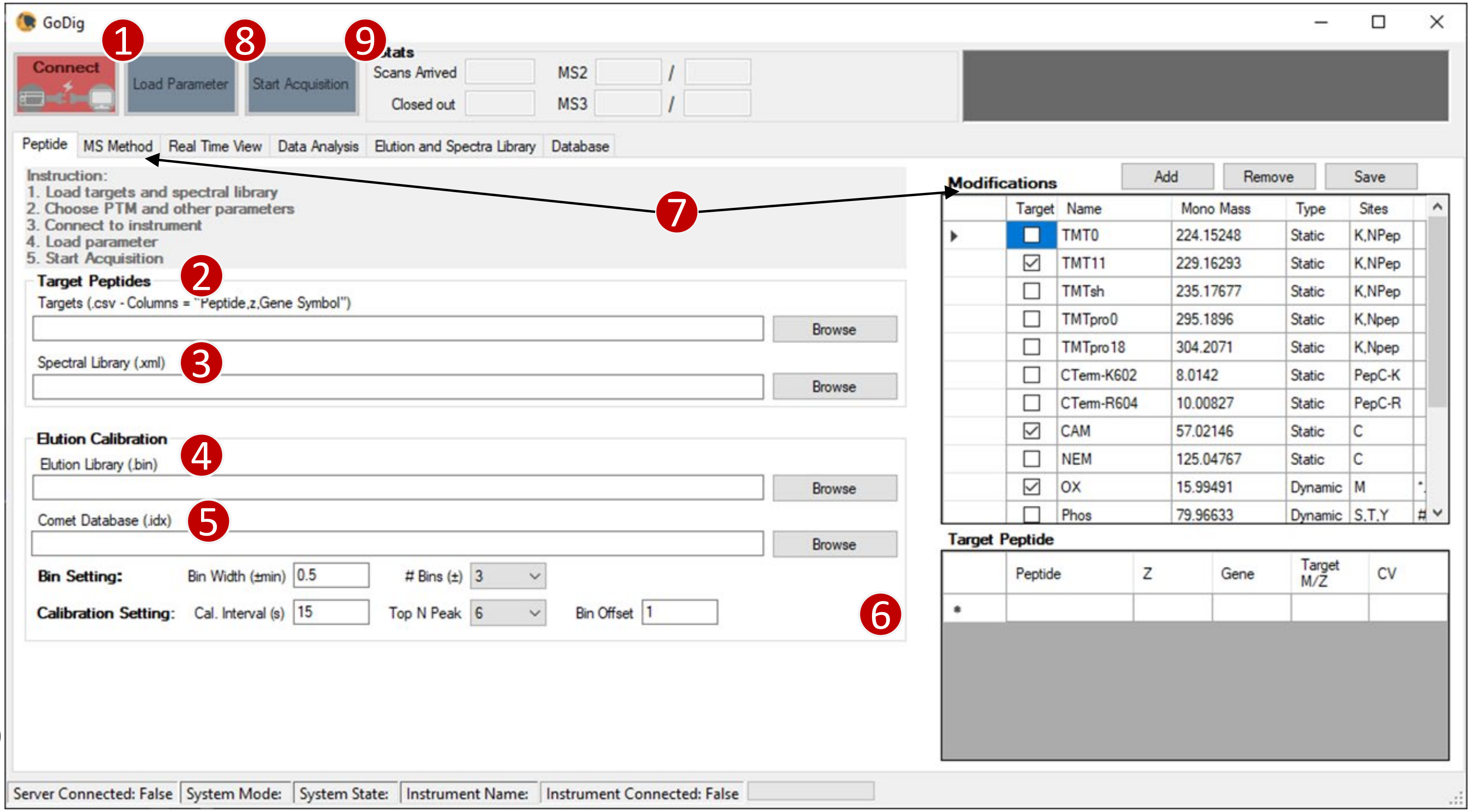

3. Select peptide spectral library (.xml)
4. Select peptide elution library
   (e.g., demo_elution_library_4_cell_line_tmtpro.bin)
5. Select proteome database
   (.idx; e.g., demo_uniprot_HUMAN_TrypsinKR_TMTPro.fasta.idx; see next slide about how to build one)
6. Select elution calibration interval and desired bin width
7. Select peptide modification and data acquisition parameters (see next slide)
8. Load parameter. This may take a couple minutes.
9. Start acquisition

How to acquire data using GoDig.exe

1. Select scheduling method (default to elution order)
2. Set dynamic exclusion (how frequent a target should be monitored)
3. Set trigger method (default to PRM)
4. Set MS parameters
5. Choose directory to write log file
6. Choose close-out option if needed

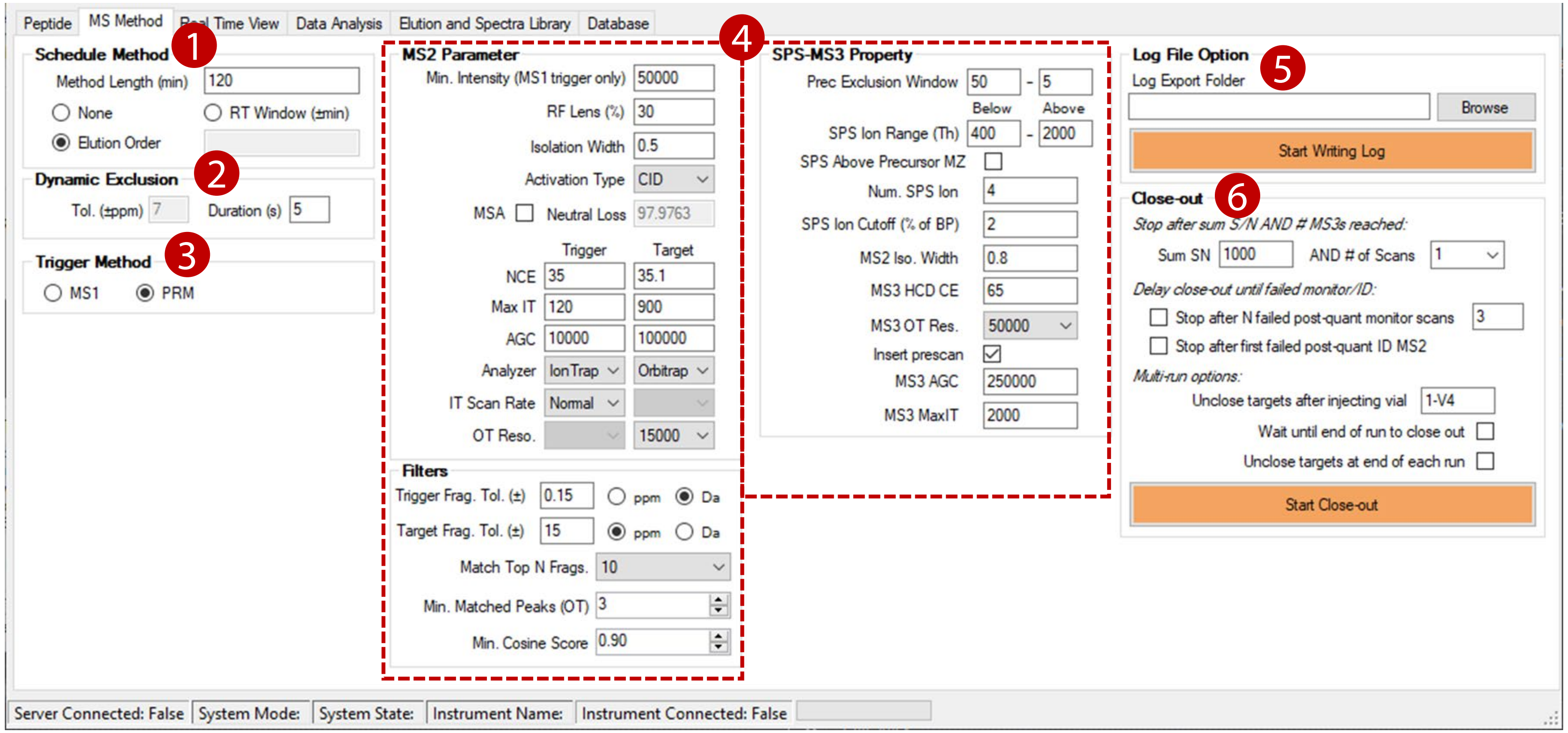

How to index a proteome .fasta file to be used for elution calibration

1. Select proteome fasta file
(can be obtained from Uniprot https://www.uniprot.org/)
2. Select TMT labeling
3. Select search parameters. GoDig uses Comet as its real-time search engine. Please refer to Comet website for all details regarding the parameters (https://comet-ms.sourceforge.net/parameters/)
4. Index database. A .idx file will be generated in the same folder as the .fasta

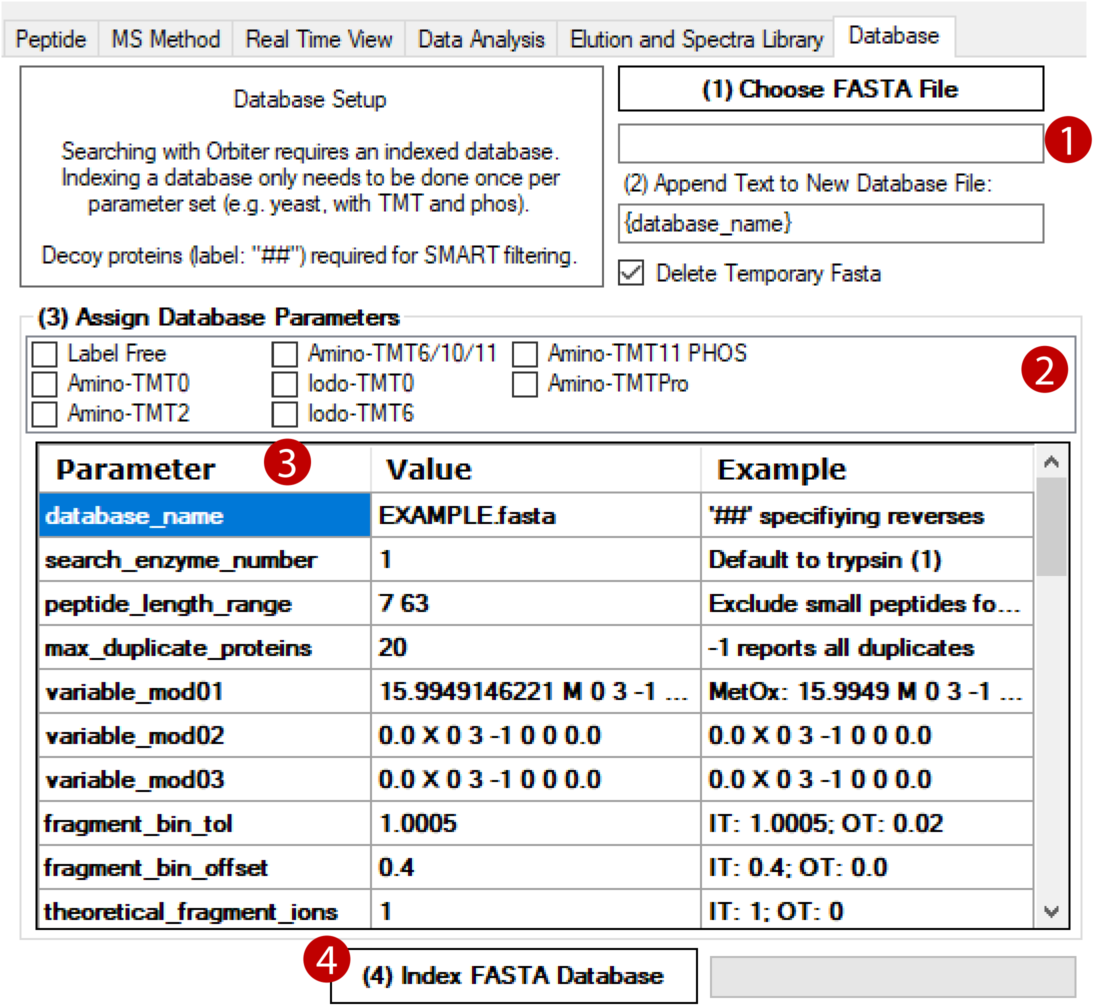

Data analysis

1. Select peptide list (.csv) and raw file. ______ you have to also select same modification, and fragment match tolerance in the method editor as the rawfile was acquired with.
2. Select raw file (.raw)
3. Select spectra library (.xml)
4. Process
5. Export to .csv

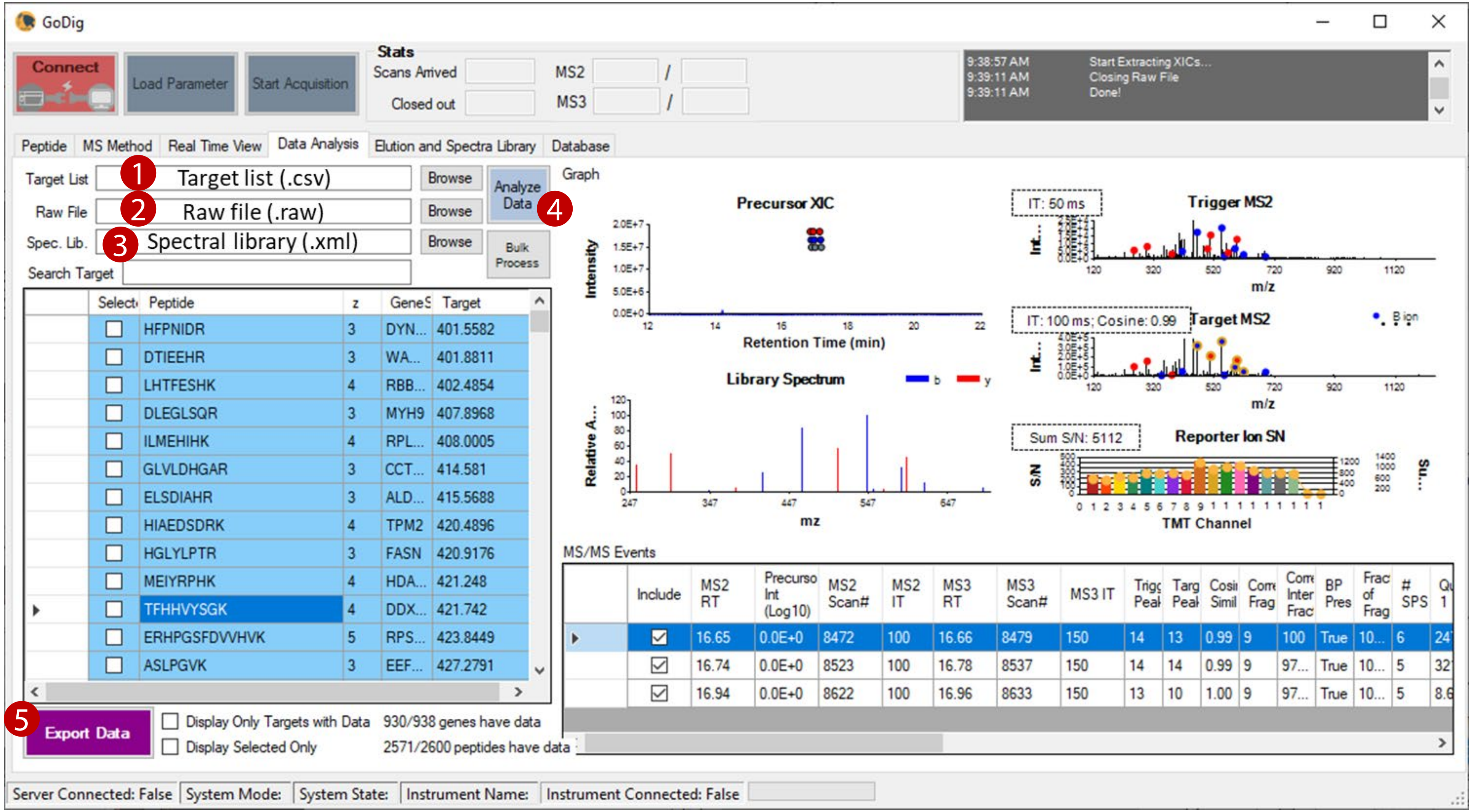

How to build custom library

1. Select peptide modification
2. Select which library to build (e.g., spectral and elution)
3. Select the .csv file that contains all identification information to be used for the library (see next slide for details)
4. Additional filters to remove low score peptides or peptide with large elution window. Fragment mass tol need to be set to build spectral library.
5. Raw files that are used to generate the .csv search result
6. Start to build (this may take a while, depending on the size of the dataset)

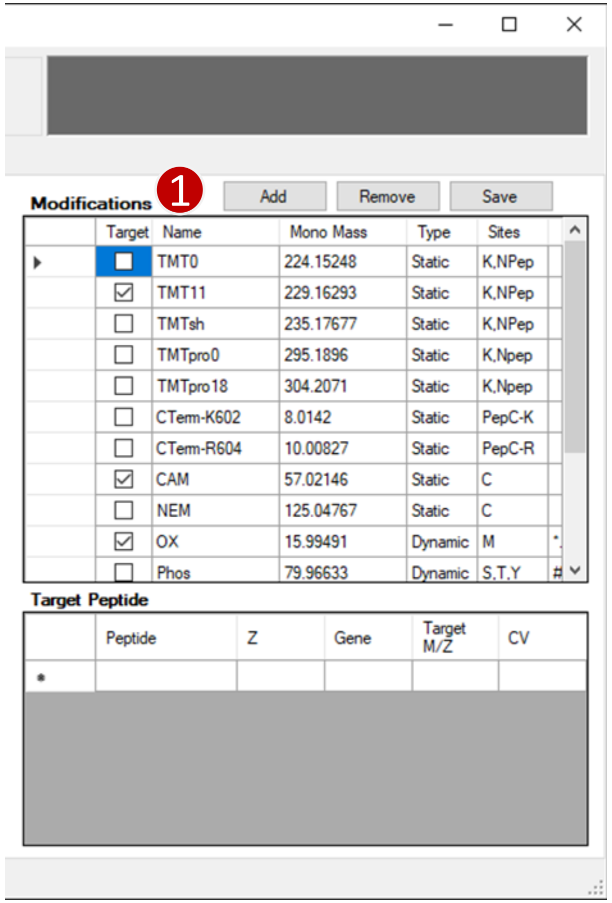

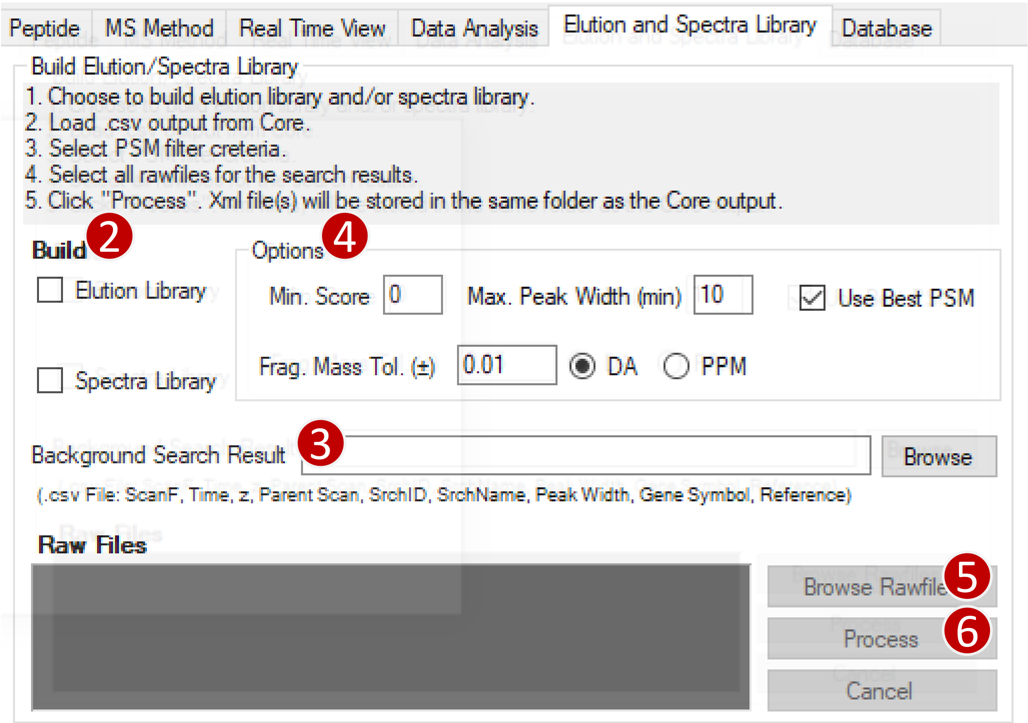

How to build custom library--.csv file

Example csv input:

Peptide	z	Theo m/z	Parent Scan	Reference	ScanF	Expect	Time	SrchID	SrchName	Peak Width	Gene Symbol	PepID
R.QQEGESR.L	2	569.2946	786	sp|P00338-3|LDHA_HUMAN	795	6.592	5.3	540183	ec04297_qy_GoDig_4cell_CIDhrMS2	0.3003	LDHA	43
R.SQEAGGR.V	2	504.7733	786	sp|P48509|CD151_HUMAN	800	5.252	5.31	540183	ec04297_qy_GoDig_4cell_CIDhrMS2	0.3003	CD151	48
K.AGQGSGSR.K	2	512.2787	794	sp|A4FU01|MTMRB_HUMAN	808	6.686	5.33	540183	ec04297_qy_GoDig_4cell_CIDhrMS2	0.3093	MTMR11	55
K.NQDDADR.A	2	569.2764	794	sp|O75937|DNJC8_HUMAN	813	7.759	5.33	540183	ec04297_qy_GoDig_4cell_CIDhrMS2	0.287	DNAJC8	60
K.REQAEEER.Y	3	450.9026	817	sp|Q9UII2|ATIF1_HUMAN	826	5.471	5.36	540183	ec04297_qy_GoDig_4cell_CIDhrMS2	0.2783	ATP5IF1	71
K.RAQEEAER.L	3	431.5674	817	tr|E7EQR4|E7EQR4_HUMAN	827	6.342	5.36	540183	ec04297_qy_GoDig_4cell_CIDhrMS2	0.3003	EZR	72
K.NEEDEGHSNSSPR.H	3	587.9368	817	sp|Q14103|HNRPD_HUMAN	830	11.026	5.36	540183	ec04297_qy_GoDig_4cell_CIDhrMS2	0.2565	HNRNPD	75
R.CNDQDTR.T	2	606.7835	817	sp|P02751-15|FINC_HUMAN	833	8.64	5.37	540183	ec04297_qy_GoDig_4cell_CIDhrMS2	0.2565	FN1	78
K.NASCGTR.S	2	535.2726	825	sp|P60468|SC61B_HUMAN	838	4.764	5.37	540183	ec04297_qy_GoDig_4cell_CIDhrMS2	0.3093	SEC61B	82

Peptide: peptide sequence with or without two flanking residues (e.g., R.QQEGESR.L or QQEGESR)
z: peptide charge state
Theo m/z: theoretical peptide m/z
Parent Scan: master scan the PSM is based on
Reference: Uniprot reference
ScanF: Scan number of the PSM
Expect: Expect score from Comet search or XCorr from Sequest search (Can be other score too but need to use the same column header)
Time: Scan time
SrchName: raw file name without the file suffix (e.g., ec04297_qy_GoDig_4cell_CIDhrMS2 from ec04297_qy_GoDig_4cell_CIDhrMS2.raw)
Peak Width: peptide peak width
Gene Symbol: gene symbol
PepID: each PSM from one raw file has a unique peptide ID. If not provided by a search engine, **ScanF** can be used instead since it is unique in each raw file.

More questions?

Please email Qing Yu (qing_yu2@hms.harvard.edu) or Steven Gygi (steven_gygi@hms.harvard.edu)
