# Supplementary material for: Sample multiplexing-based targeted pathway proteomics with real-time analytics reveals the impact of genetic variation on protein expression
Source: Nat Commun. 2023 Feb 2;14:555. doi: 10.1038/s41467-023-36269-7 (PMC9894840; doi:10.1038/s41467-023-36269-7)
Supplement: Supplementary file 2 — Description of Additional Supplementary Files [file 41467_2023_36269_MOESM2_ESM.docx]

**Supplementary Data 1.** Scaled protein abundance in 4 human cell lines. Related to Fig. 2, 3 and Supplementary Fig. 4.

**Supplementary Data 2.** Scaled protein abundance in 8 founder strains. Data were acquired with real-time search. Related to Fig. 4-7 and Supplementary Fig. 5-7.

**Supplementary Data 3.** Protein abundance in 480 DO livers quantified using GoDig. Related to Fig. 4-7 and Supplementary Fig. 6-8.

**Supplementary Data 4.** Identified pQTLs with GoDig targeted analysis. Related to Fig. 4-7 and Supplementary Fig. 7-8.

**Supplementary Data 5.** Mediation analysis to include protein abundance as covariates in lipid QTL scanning. Related to Fig. 6, 7 and Supplementary Fig. 8.

**Supplementary Data 6.** Target peptides used in GoDig experiments.

**Supplementary Data 7.** Peptide information used to build the 4 cell line library. Related to Fig. 2, 3 and Supplementary Fig. 3.

**Source Data.** Source data for plots.
