# Supplementary material for: Sample multiplexing-based targeted pathway proteomics with real-time analytics reveals the impact of genetic variation on protein expression
Source: Nat Commun. 2023 Feb 2;14:555. doi: 10.1038/s41467-023-36269-7 (PMC9894840; doi:10.1038/s41467-023-36269-7)
Supplement: Supplementary file 10 — Reporting Summary [file 41467_2023_36269_MOESM10_ESM.pdf]

nature portfolio

Corresponding author(s): Steven Gygi

Last updated by author(s): Dec 28, 2022

Reporting Summary

Nature Portfolio wishes to improve the reproducibility of the work that we publish. This form provides structure for consistency and transparency in reporting. For further information on Nature Portfolio policies, see our Editorial Policies and the Editorial Policy Checklist.

Statistics

For all statistical analyses, confirm that the following items are present in the figure legend, table legend, main text, or Methods section.

n/a	Confirmed	
☐	☒	The exact sample size (*n*) for each experimental group/condition, given as a discrete number and unit of measurement
☐	☒	A statement on whether measurements were taken from distinct samples or whether the same sample was measured repeatedly
☐	☒	The statistical test(s) used AND whether they are one- or two-sided<br>*Only common tests should be described solely by name; describe more complex techniques in the Methods section.*
☐	☒	A description of all covariates tested
☐	☒	A description of any assumptions or corrections, such as tests of normality and adjustment for multiple comparisons
☐	☒	A full description of the statistical parameters including central tendency (e.g. means) or other basic estimates (e.g. regression coefficient) AND variation (e.g. standard deviation) or associated estimates of uncertainty (e.g. confidence intervals)
☐	☒	For null hypothesis testing, the test statistic (e.g. *F*, *t*, *r*) with confidence intervals, effect sizes, degrees of freedom and *P* value noted<br>*Give P values as exact values whenever suitable.*
☒	☐	For Bayesian analysis, information on the choice of priors and Markov chain Monte Carlo settings
☒	☐	For hierarchical and complex designs, identification of the appropriate level for tests and full reporting of outcomes
☐	☒	Estimates of effect sizes (e.g. Cohen's *d*, Pearson's *r*), indicating how they were calculated

Our web collection on statistics for biologists contains articles on many of the points above.

Software and code

Policy information about availability of computer code

Data collection	For unbiased discovery proteomics data, mass spectra were acquired using a Orbitrap Eclipse instrument from Thermo Fisher Scientific with the corresponding software provided by the vendor. On-line real-time searching was done via Orbiter version 1.7 (https://pubs.acs.org/doi/abs/10.1021/acs.jproteome.9b00860). The Orbiter software is available via a free user license for the Orbitrap Eclipse mass spectrometer platform with an API license (https://gygi.hms.harvard.edu/software.html).<br><br>For targeted proteomics data, we used custom code to allow GoDig (v.1.0.0) to perform the designed experiment. To enable GoDig, the real-time search Comet functionality is required and has already been released (http://comet-ms.sourceforge.net/). The GoDig software is available via a free user license for the Orbitrap Eclipse mass spectrometer platform with an API license (https://gygi.hms.harvard.edu/software.html).
Data analysis	Data analysis were performed in R (v. 4.0.5) using RStudio. R packages used include tidyr (v. 1.1.3), ggplot2 (v. 3.3.5), tibble (v. 3.1.2), qtl2 (v. 0.24),intermediate (v. 2.5), qvalue (v. 2.22.0), stringr (v. 1.4.0) and RColorBrewer (v.1.1)

For manuscripts utilizing custom algorithms or software that are central to the research but not yet described in published literature, software must be made available to editors and reviewers. We strongly encourage code deposition in a community repository (e.g. GitHub). See the Nature Portfolio guidelines for submitting code & software for further information.

Data

Policy information about availability of data

All manuscripts must include a data availability statement. This statement should provide the following information, where applicable:

- Accession codes, unique identifiers, or web links for publicly available datasets
- A description of any restrictions on data availability
- For clinical datasets or third party data, please ensure that the statement adheres to our policy

This project has generated many types of data and code that are available for distribution via numerous venues. Items not listed here will be provided by the Lead Contact upon reasonable request.

The mass spectrometry data profiling liver proteomes from 8 mouse founder strains have been deposited at the ProteomeXchange Consortium with the dataset identifier PXD029461 (https://www.ebi.ac.uk/pride/archive/projects/PXD029461). Global proteome data in founder mouse strains generated during this study are also available using the viewer on the Gygi lab website (https://gygi.hms.harvard.edu/resources.html).

The mass spectrometry data profiling 4 human cell lines have been deposited at the ProteomeXchange Consortium with the dataset identifier PXD033643 (https://www.ebi.ac.uk/pride/archive/projects/PXD033643).

The mass spectrometry data profiling AML12WT and AML12GM4951 with and without siGM4951 have been deposited at the ProteomeXchange Consortium with the dataset identifier PXD033679 (https://www.ebi.ac.uk/pride/archive/projects/PXD033679).

The 297 raw files from GoDig experiments have been deposited at the MassIVE repository (ftp://massive.ucsd.edu) with the dataset identifier MSV000090110 (ftp://massive.ucsd.edu/MSV000090110/).

The liver lipidomics dataset used here was published by Linke et al and has been deposited in Chorus (https://chorusproject.org/anonymous/download/experiment/a639bcc5602c441c9a1df94f4340d626)

Human research participants

Policy information about studies involving human research participants and Sex and Gender in Research.

Reporting on sex and gender	N/A
Population characteristics	N/A
Recruitment	N/A
Ethics oversight	N/A

Note that full information on the approval of the study protocol must also be provided in the manuscript.

Field-specific reporting

Please select the one below that is the best fit for your research. If you are not sure, read the appropriate sections before making your selection.

☒ Life sciences ☐ Behavioural & social sciences ☐ Ecological, evolutionary & environmental sciences

For a reference copy of the document with all sections, see nature.com/documents/nr-reporting-summary-flat.pdf

Life sciences study design

All studies must disclose on these points even when the disclosure is negative.

Sample size

For the 8 founder strain mice, 64 founder mice (8 per strain [4 male & 4 female]) were used to maximize efficiency and balance of samples based on sex and strain across TMT 16plexes, allowing for 4 replicates of each strain-by-sex combination. N = 4 per group (sex and strain) provides 90% power at alpha = 0.05 to detect a change of 2 standard deviations (SD), where SD refers the measurement error of TMT-based proteomics. In light of the precision of the technology we deemed this sufficient to detect most biologically meaningful changes. Comparisons pooled across sex, or contrasts between sets of strains to compare genetic effects will have greater power.

The key quantity for sample size estimation in the DO mouse experiment is the percent of total variance explained by the genotype at a locus, where percent variance explained is the change in phenotype due to allelic substitution times the allele frequency. Gatti et al. (2014, G3) present simulation results for DO mouse mapping based on stringent genome-wide adjusted significance levels (Figure 4 in Gatti et al. 2014, G3). Based on this, we determined that for n approaching 500 mice, it should be possible to detect a locus that explains 10% of total variance at power greater than 0.80. Note, percent variance explained is typically much higher in the DO mouse population in comparison to human populations, due to the absence of rare allele in the DO mice. A typical locus associated with gene expression or protein abundance can explain 20% to 80% of the total variance. As such, the study is well powered to detect loci with very subtle effects on protein abundance.

Data exclusions	No exclusion criteria were pre-established. Data quality was determined by calculating the summed signal-to-noise of each peptide used in analysis.
Replication	All attempts at replication were successful and consistent in at least two independent experiments.
Randomization	For the Diversity Outbred mouse cohort, the mice were assigned to each TMTpro16 plex so that each TMTpro16 set comprises equal number of male and female mice to reach a balanced setup between sexes. Each of the DO mice is genetically unique, therefore, no further randomization is needed.<br><br>For the 8 founder strain mice, 64 founder mice (8 per strain [4 male & 4 female]) were used to maximize efficiency and balance of samples based on sex and strain across TMT 16plexes, allowing for 4 replicates of each strain-by-sex combination.
Blinding	Investigators were not blinded to sample allocation in TMT experiments. Blinding was not relevant because no human subjective qualitative results were reported.

Reporting for specific materials, systems and methods

We require information from authors about some types of materials, experimental systems and methods used in many studies. Here, indicate whether each material, system or method listed is relevant to your study. If you are not sure if a list item applies to your research, read the appropriate section before selecting a response.

Materials & experimental systems

n/a	Involved in the study
☒	☐ Antibodies
☐	☒ Eukaryotic cell lines
☒	☐ Palaeontology and archaeology
☐	☒ Animals and other organisms
☒	☐ Clinical data
☒	☐ Dual use research of concern

Methods

n/a	Involved in the study
☒	☐ ChIP-seq
☒	☐ Flow cytometry
☒	☐ MRI-based neuroimaging

Eukaryotic cell lines

Policy information about cell lines and Sex and Gender in Research

Cell line source(s)	All four human cell lines (U2OS, RPE1, HCT116, HEK293T, AML12) were obtained from ATCC.
Authentication	Fresh cells were purchased directly from ATCC and propagated for use. No additional authentication was performed in-house.
Mycoplasma contamination	Low passage cell stocks were confirmed mycoplasma negative by PCR upon arrival from ATCC.
Commonly misidentified lines<br>(See ICLAC register)	No commonly misidentified lines were used.

Animals and other research organisms

Policy information about studies involving animals; ARRIVE guidelines recommended for reporting animal research, and Sex and Gender in Research

Laboratory animals	480 diversity outbred mice (240 female and 240 male) and 63 mice (32 female and 31 male) from 8 founder strains (A/J, C57BL/6J, CAST/EiJ, NOD/ShiLtJ, NZO/H1LtJ, PWK/PhJ, WSB/EiJ, 129S1/SvlmJ) were included in the study. The mice were obtained at 4 weeks of age and maintained within the Department of Biochemistry animal vivarium at the University of Wisconsin. All DO mice were maintained on a HF/HS (high fat/ high sucrose) diet (44.6% kcal fat, 34% carbohydrate, and 17.3% protein) from Envigo Teklad (catalog number TD.08811). All mice were maintained in a temperature and humidity-controlled room on a 12h light/dark cycle (lights on at 6:00 and off at 18:00), and provided water ad libitum. At ~22 weeks of age, DO mice were euthanized with CO2, and their livers were harvested and flash frozen in LN2.
Wild animals	The study does not involve wild animals.
Reporting on sex	Equal number of animals of both sexes were included in the study and mouse protein abundance was modeled with linear regression with strain and sex as covariates. For QTL mapping, sex and batch (breeding generation of the mice) were included as additive covariates and a random polygenic term to account for genetic relatedness was included in the mapping
Field-collected samples	The study does not involve field-collected samples.
Ethics oversight	All experiments involving mice were preapproved by an AAALAC-accredited Institutional Animal Care and Use Committee of the

Ethics oversight	College of Agricultural Life Sciences (CALS) at the University of Wisconsin–Madison. The CALS Animal Care and Use Protocol number associated with the study is A005821, A.D. Attie, Principal Investigator.

Note that full information on the approval of the study protocol must also be provided in the manuscript.
